# Controllable stereoinversion in DNA-catalyzed olefin cyclopropanation *via* cofactor modification†

**DOI:** 10.1039/d1sc00755f

**Published:** 2021-05-11

**Authors:** Jingya Hao, Wenhui Miao, Shengmei Lu, Yu Cheng, Guoqing Jia, Can Li

**Affiliations:** State Key Laboratory of Catalysis, Dalian Institute of Chemical Physics, Chinese Academy of Sciences Zhongshan Road 457 Dalian 116023 China canli@dicp.ac.cn; University of Chinese Academy of Sciences Beijing 101408 China

## Abstract

The assembly of DNA with metal-complex cofactors can form promising biocatalysts for asymmetric reactions, although catalytic performance is typically limited by low enantioselectivities and stereo-control remains a challenge. Here, we engineer G-quadruplex-based DNA biocatalysts for an asymmetric cyclopropanation reaction, achieving enantiomeric excess (ee_trans_) values of up to +91% with controllable stereoinversion, where the enantioselectivity switches to −72% ee_trans_ through modification of the Fe-porphyrin cofactor. Complementary circular dichroism, nuclear magnetic resonance, and fluorescence titration experiments show that the porphyrin ligand of the cofactor participates in the regulation of the catalytic enantioselectivity *via* a synergetic effect with DNA residues at the active site. These findings underline the important role of cofactor modification in DNA catalysis and thus pave the way for the rational engineering of DNA-based biocatalysts.

## Abstract 2

Cofactor modification in a DNA-catalyzed olefin cyclopropanation reaction enables controllable stereoinversion and achieves enantioselectivities of up to +91% and −72% ee_trans_.

## Graphical abstract

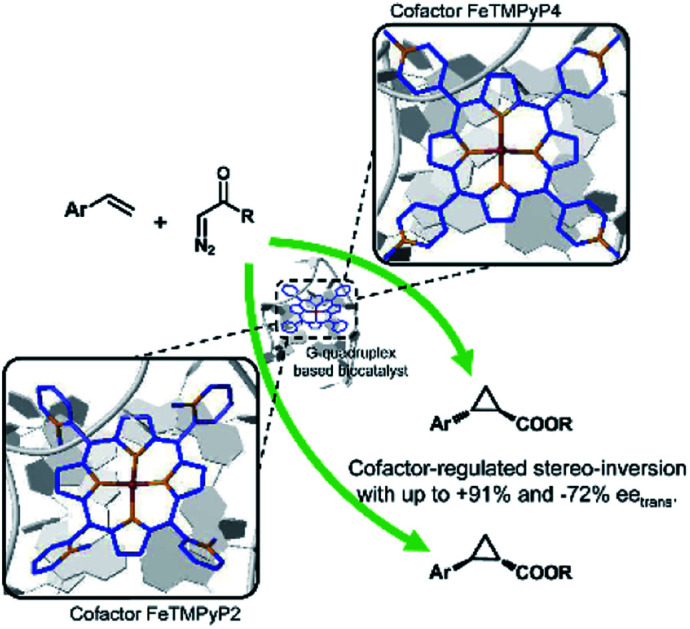

## Introduction

Cyclopropane motifs feature in many natural products and medicinal agents^1^ which constitute versatile intermediates for the total synthesis of therapeutic compounds.^2^ As these compounds are in high demand, significant effort has been devoted to the development of cyclopropane synthesis, in particular *via* the use of hemoprotein enzymes engineered *via* directed evolution. Cytochrome P450 (ref. 3 and ^4^) and myoglobin^5–8^ enzymes have been evolved to catalyze asymmetric olefin cyclopropanations with excellent performance.^9–14^ This method has been applied to the synthesis of drug molecules^15,16^ and natural product scaffolds.^17,18^

Despite this progress, the developed biocatalytic protocols are generally restricted to protein enzyme engineering.^19^ The discovery of the catalytic functions of nucleic acids^20,21^ has expanded the breadth of biocatalytic protocols to include RNA and DNA catalysts, initiating the pursuit of nucleic acid–based enzymes. DNA possesses inherent advantages as a catalyst. A catalytic sequence can be entirely identified from a random sequence population and folds into its practical tertiary structure spontaneously. A number of asymmetric reactions, especially Lewis acid-catalyzed reactions, have been successfully realized using DNA-based biocatalysts resulting in remarkable performances.^22–31^ Recently, the Roelfes^32^ and Sen^33^ groups expanded the scope of reactions catalyzed by DNA-based biocatalysts to include olefin cyclopropanation. Although the enantioselectivities achieved were moderate, this has paved the way for DNA-catalyzed carbene transfer reactions. Therefore, designing a DNA-based biocatalyst that catalyzes cyclopropanation in high enantiomeric excess (ee) remains a challenge, especially to achieve an ee greater than 90%.^34^

DNA catalysis is opening a promising avenue for biosynthesis, but the catalytic scope and performance of DNA catalysts still need improvement in comparison with catalysts based on protein enzymes. Considering the great role played by the cofactor ligand in the first-coordination-sphere in catalytic reactions,^35–40^ cofactor modification, which is as powerful as directed evolution^41–44^ but seriously disregarded in the field of DNA catalysis, is introduced for the development of DNA-based biocatalysts. Here, we report a cyclopropanation reaction catalyzed by a G-quadruplex (G4)–Fe-porphyrin biocatalyst that results in enantioselectivity as high as 91%. By tuning the *N*-methyl position of Fe-porphyrin from the *para*- to the *ortho*-position, the catalytic enantioselectivity of the reconstituted G4–Fe-porphyrin biocatalyst reverses to −72% ee_trans_. Complementary spectral, nuclear magnetic and isothermal titration characterization studies reveal that the stereo-divergence of the product mainly arises from the participation of the porphyrin ligand in the regulation of the enantioselectivity. This work succeeds in diversifying the functionality of DNA-based biocatalysts *via* cofactor modification, highlighting the great potential for cofactor modification in the field of DNA-based biocatalyst engineering.

## Results and discussion

**Fig. 1:**
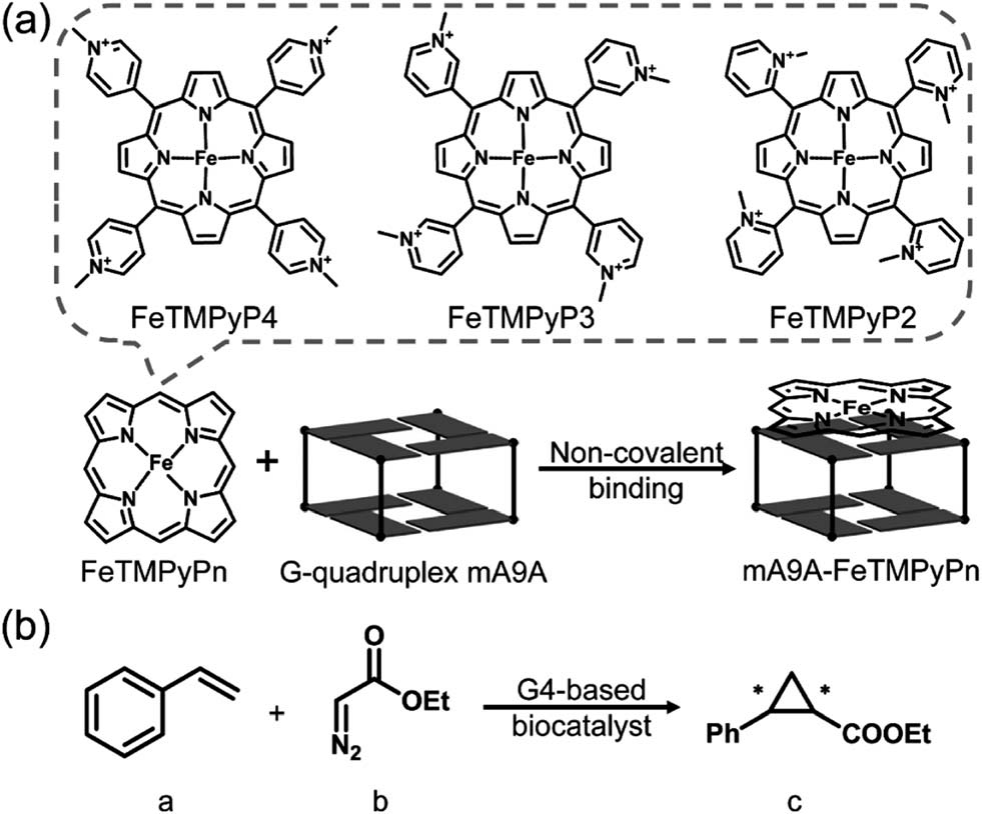
(a) The assembly of the G4-based biocatalysts. (b) The asymmetric cyclopropanation reaction investigated in this work.

Fig. 1a illustrates the design of the G4-based biocatalysts. Three Fe-*meso*-tetra-(*N*-methylpyridyl)porphyrins with *para*- (FeTMPyP4), *meta*- (FeTMPyP3), and *ortho*- (FeTMPyP2) *N*-methyl substituents were chosen as parallel cofactors to investigate the effect of the first-coordination-sphere on the catalytic performance. The non-covalent binding of the cofactors with mA9A G4 (d[G_2_T_2_G_2_TGAG_2_T_2_G_2_A]), a thrombin binding aptamer (TBA) variant, formed the G4-based biocatalysts. The assembled biocatalysts were then tested in a reaction between styrene and ethyl diazoacetate (EDA), which results in a chiral cyclopropane product (Fig. 1b).

Table 1 lists the results obtained using the Fe-porphyrins and their corresponding G4 biocatalysts. The use of the free FeTMPyP*n* (*n* = 4, 3, 2) cofactor as the catalyst led to relatively low activities and no chiral induction (Table 1, entries 1–3). The biocatalysts (mA9A-FeTMPyP*n*, *n* = 4, 3, 2) assembled from FeTMPyP*n* and mA9A G4 significantly improved the catalytic activities with turnover frequencies (TOF) increased about 10-fold when compared to those of the free FeTMPyP*n*s. More surprisingly, mA9A-FeTMPyP2 induces the inversion of the enantioselectivity relative to that of mA9A-FeTMPyP4 from +74% to −46% (Table 1, entries 4–6). To further understand the complementary chiral induction mechanism, circular dichroism (CD), nuclear magnetic resonance (NMR), fluorescence titration and other characterization methods were then performed.

**Table 1:** Activities and selectivities of FeTMPyP*n* and mA9A-FeTMPyP*n* (*n* = 4, 3, 2) in the cyclopropanation reaction of styrene with EDA^a^

Entry	Catalyst	% product conversion	TOF (h^−1^)	% ee_trans_^b^
1	FeTMPyP4	5	16	0
2	FeTMPyP3	6	20	0
3	FeTMPyP2	4	11	0
4	mA9A-FeTMPyP4	46	184	74
5	mA9A-FeTMPyP3	36	144	63
6	mA9A-FeTMPyP2	27	108	−46

a The experiments were carried out with styrene (30 mM), EDA (10 mM), DNA (12.5 μM), and FeTMPyP*n* (*n* = 2, 3, 4) (12.5 μM) in 10 mM potassium phosphate buffer (pH 7.0) under argon at 4 °C for 2 h, unless otherwise specified. Product conversions and ee values are based on the areas of HPLC peaks as compared to an internal standard. The diastereomeric ratios of the products ranged from 90 : 10 to 97 : 3. All data are the average of 3 attempts, reproducibility ±5%.

b (*R*,*R*)–(*S*,*S*).

**Fig. 2:**
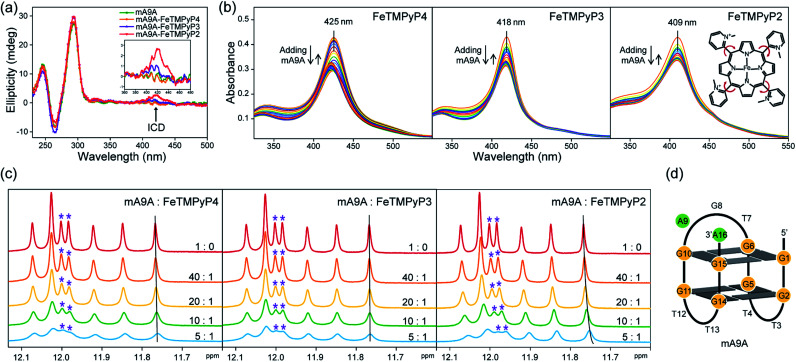
(a) CD spectra of mA9A and mA9A-FeTMPyP*n* (*n* = 4, 3, 2) (inset: a detailed view of the ICD signals). (b) UV-vis absorption spectra of FeTMPyP*n* (*n* = 4, 3, 2) when titrated with mA9A. (c) NMR spectra of mA9A over the range of 11.5–12.5 ppm when titrated with FeTMPyP*n* (*n* = 4, 3, 2). (d) The G-quadruplex structure of mA9A.

The CD spectra in Fig. 2a show that the antiparallel G4 signatures of mA9A, featuring two positive peaks at 245 nm and 295 nm, and one negative peak at 265 nm, are still present in the mA9A-FeTMPyP*n* (*n* = 4, 3, 2) catalysts. But of note is that the CD spectrum of mA9A-FeTMPyP2 shows a specifically induced CD signal (ICD) at 420 nm. Only when an asymmetric conformation is formed can an ICD signal be induced. DNA does not absorb at 420 nm, but FeTMPyP2 does (Fig. 2b). Therefore, the ICD signal can be attributed to FeTMPyP2, whose planar symmetry is broken due to the interaction with mA9A.

The ultraviolet-visible (UV) absorption spectra (Fig. 2b) of mA9A-FeTMPyP*n* further support the CD results. The spectra of FeTMPyP*n* (*n* = 4, 3, 2) exhibit broad Soret absorption bands at around 420 nm. As the *N*-methyl group varies from the *para*- to the *ortho*-position, the Soret band shows a blue shift, indicating that FeTMPyP2 has decreased electronic conjugation relative to the other porphyrins. Due to the steric hindrance of the 2-*N*-methyl group, the bond between the pyridine ring and the porphyrin ring of FeTMPyP2 has a tendency to rotate, causing the pyridine ring and the porphyrin ring to be non-co- planar (Fig. 2b, right inset), which explains the generation of an ICD signal in mA9A-FeTMPyP2. When well-folded mA9A is added to FeTMPyP*n*, the Soret bands first fall to a minimum and then slightly rise for all cofactors. This suggests that FeTMPyP*n* (*n* = 4, 3, 2) have a similar external stacking mode on mA9A, rather than intercalating between two G-quartets.^45–47^ Moreover, binding with FeTMPyP4 and FeTMPyP3 makes mA9A more stable according to UV melting experiments (Fig. S1†), while binding with FeTMPyP2 does not.

To obtain further structural information on the mA9A-FeTMPyP*n* catalysts, we conducted an NMR characterization of the systems. The NMR spectrum of mA9A shows seven discrete peaks over the range of 11.5–12.5 ppm (Fig. 2c), which can be assigned to the eight guanine imino protons (H1) of the G-quartet. These signals indicate the formation of a two-layer antiparallel G4 structure in the potassium phosphate buffer (Fig. 2d), like that of TBA.^48,49^ NMR titration experiments (Fig. 2c) where mA9A is added to the three FeTMPyP*n* (*n* = 4, 3, 2) cofactors show similar line broadening and reductions in peak intensity. However, there are two adjacent peaks around 12.0 ppm (peaks labelled with *) with different relative declines, indicating that the coordination modes between mA9A and the different Fe-porphyrin cofactors vary slightly. Moreover, the addition of FeTMPyP2 causes an obvious upfield peak-shift, which is attributed to the strong electronic shielding effect arising from the porphyrin ligand stacking upon the G-quartet.^50^ Therefore, in combination with the CD and UV-vis results, we find that FeTMPyP2 adjusts to an optimal conformation through bond rotation and distortion, thereby forming a tight π–π stacking mode with mA9A and creating a strong electronic shielding effect on the imino protons of the G-quartet.

**Fig. 3:**
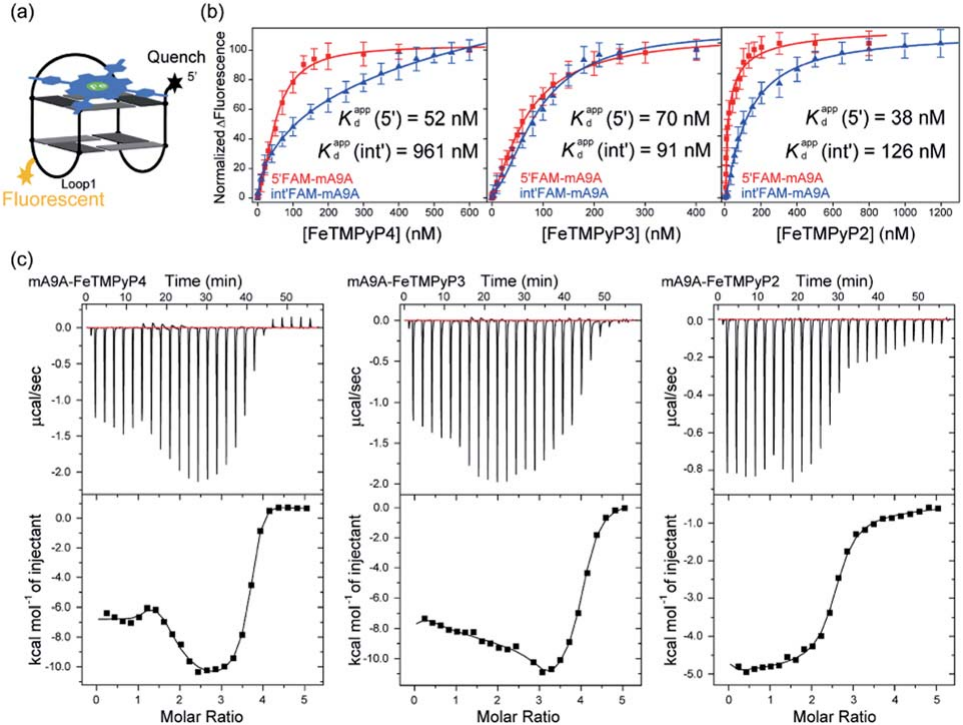
(a) A schematic diagram of the fluorescence quenching equilibrium dissociation binding assay. (b) Binding curves determined using fluorescence quenching titration. (c) ITC profiles for the binding of FeTMPyP*n* (*n* = 4, 3, 2) with mA9A.

A fluorescence-based binding assay was then implemented to locate the catalytic sites.^51^ Since UV titration had determined the external stacking mode of the FeTMPyP*n* (*n* = 4, 3, 2) cofactors on mA9A, the 5′ end (5′FAM-mA9A) and loop 1 (int′FAM-mA9A) of the two G-quartets of mA9A were labelled separately with the fluorophore 5-carboxyfluorescein (FAM). The fluorescence of FAM can be suppressed by the presence of FeTMPyP*n* (Fig. 3a and S2†). By adding FeTMPyP*n* dropwise to the FAM-labelled mA9A, we obtained the titration quenching curves (Fig. S3†). The fitted apparent equilibrium dissociation constants (*K*d^app^) at the different FAM-labelled sites, shown in Fig. 3b, indicate that all the FeTMPyP*n* (*n* = 4, 3, 2) cofactors prefer binding at the 5′, 3′ end of the G-quartet. Considering that the ratio of mA9A and FeTMPyP*n* is greater than 1 : 1 when assembling the DNA-based biocatalyst and that the ee_trans_ values for the catalytic cyclopropanation can be maintained at the highest level, the preferential binding site of FeTMPyP*n* with mA9A is regarded as the active center for chiral regulation. Therefore, according to the fluorescence-based binding assay, FeTMPyP*n* binding at the 5′, 3′-end of the G-quartet of mA9A constructs the active catalytic site of mA9A-FeTMPyP*n*. The *K*d^app^ of FeTMPyP2 is the lowest at 38 nM, compared to 70 nM for FeTMPyP3 and 52 nM for FeTMPyP4, indicating that FeTMPyP2 binds the strongest with mA9A.

Isothermal titration calorimetry (ITC) provides information on intermolecular interactions by recording the heat discharged or consumed during a bimolecular reaction. Fig. 3c shows that there are several distinct exothermic processes during the titration of mA9A with FeTMPyP4 and FeTMPyP3, indicating complicated multiple binding behaviours between FeTMPyP4, FeTMPyP3 and mA9A. As explained in the above section, although mA9A enables the binding of multiple iron porphyrins, the strongest binding sites were the catalytic sites for the chiral regulation of the cyclopropanation reaction. The titration curves were fitted using a sequential binding sites model to calculate the binding parameters. The highest affinities between FeTMPyP*n* and mA9A were quantified to be 33 nM (*n* = 4), 50 nM (*n* = 3) and 14 nM (*n* = 2), coinciding with the trend of *K*d^app^ as measured using fluorescence titration. This further supports the conclusion that the 5′, 3′-end of the G-quartet of mA9A constructs the active catalytic sites. Given that FeTMPyP2 has flexible peripheral groups (according to UV spectra), the tight assembly with mA9A can be attributed to the conformational transition of the TMPyP2 ligand, which is also confirmed by the CD spectra.

**Fig. 4:**
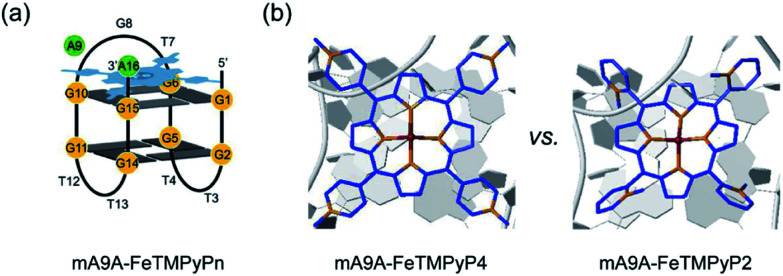
(a) A schematic diagram of the assembly of mA9A-FeTMPyP*n* (*n* = 4, 3, 2). (b) Schematic diagrams of the active centres of mA9A-FeTMPyP4 and mA9A-FeTMPyP2.

NMR, UV and fluorescence titration studies indicate that fine-tuning the *N*-methyl position of the cofactor does not change the preference of FeTMPyP*n* for binding at the 5′, 3′-end of the G-quartet of mA9A. This suggests that a similar active DNA pocket for cyclopropanation catalysis is provided by all the mA9A-FeTMPyP*n* (*n* = 4, 3, 2) catalysts (Fig. 4a). The ligands TMPyP4 and TMPyP3 are planar symmetric molecules that are unable to induce chirality in the catalytic process of the mA9A-FeTMPyP4 and mA9A-FeTMPyP3 catalysts. It is the deoxynucleotide residues at the 5′, 3′-end of the G-quartet of mA9A that hold the iron porphyrin carbene (IPC) intermediate in a certain orientation and define the configuration of the product (Fig. 4b). Nevertheless, mA9A-FeTMPyP3 shows a similar but lower enantioselectivity than mA9A-FeTMPyP4. Considering the similar binding strengths of FeTMPyP3 with the two G-quartets of mA9A (Fig. 3b), the reduction in enantioselectivity can be reasonably attributed to the multi-site binding behaviour of FeTMPyP3. In contrast to mA9A-FeTMPyP4 and mA9A-FeTMPyP3, the symmetry breaking of the porphyrin ring in TMPyP2 allows it to participate in chiral regulation. This, in synergy with the deoxynucleotide residues, induces the formation of the cyclopropane product with the opposite configuration to that catalyzed by mA9A-FeTMPyP4 (Fig. 4b).

To extend the substrate scope of the mA9A-FeTMPyP*n* catalyzed cyclopropanation reaction, a series of olefins and diazoesters were investigated (Table 2). All three mA9A-FeTMPyP*n* (*n* = 4, 3, 2) catalysts show obvious substrate specificities. The catalytic enantioselectivities of mA9A-FeTMPyP4 and mA9A-FeTMPyP3 toward olefin substrates with substituents on the phenyl group are reduced (entries 1 *vs.* 2–6). The enlargement of the diazoester functional group from ethyl (Et) to –CCH_3_(i-Pr)_2_ (i-Pr is the abbreviation for isopropyl) enables a significant enhancement in the ee_trans_ values to 91% (entries 1 *vs.* 8–10). However, the use of a –CH(Cy)_2_ (Cy is the abbreviation for cyclohexyl, entry 11) group does not result in an enhancement. Several studies have reported that the modification of diazoester substituents causes a significant impact on the activities and selectivities of carbene transfer reactions.^6,52^ The G4–Fe-porphyrin-catalyzed cyclopropanation has been characterized to proceed through a catalytic IPC intermediate.^34^ Diazoester reagents attack the active [Fe] center to form an IPC intermediate and release one molecule of N_2_. An appropriate substituent on the diazoester reagent can coordinate with the deoxynucleotide residues (especially dA9) through hydrophobic interactions to directly determine the conformation and properties of the IPC intermediate. Therefore, a –CCH_3_(i-Pr)_2_ substituent promotes IPC convergence to a single well-defined orientation, and the steric hindrance of the deoxynucleotide residues allows one face of the IPC to be exposed to the olefin, while keeping the other inaccessible, resulting in high enantioselectivity. For mA9A-FeTMPyP2, variation in the substituents on the phenyl group of the olefin increases the enantioselectivity to −72% ee_trans_ (entry 6), but the catalytic cyclopropanation activities are generally lower than those of the other two biocatalysts. Although the three mA9A-FeTMPyP*n* (*n* = 4, 3, 2) catalysts show different responses to substrate variation, they are all *trans* product-selective with *trans*/*cis* ratios of more than 86 : 14, and almost nonselective toward the R1-substituted olefin substrate (entry 7).

**Table 2:** Substrate scope for mA9A-FeTMPyP*n* (*n* = 4, 3, 2) catalyzed cyclopropanation^a^

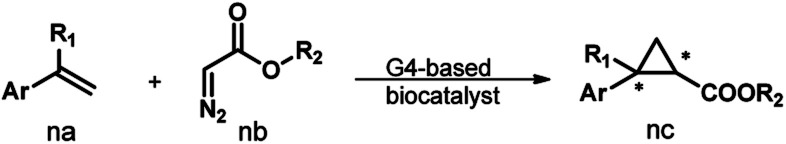										
Entry	Product	mA9A-FeTMPyP4			mA9A-FeTMPyP3			mA9A-FeTMPyP2		
		% product conversion	TOF (h^−1^)	% ee_trans_^b^	% product conversion	TOF (h^−1^)	% ee_trans_^b^	% product conversion	TOF (h^−1^)	% ee_trans_^b^
	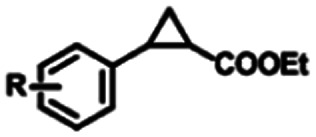									
1	1c, R = 4-H	46	184	74	36	144	63	27	108	−46
2	2c, R = 4-Me	36	144	70	35	140	60	25	100	−45
3	3c, R = 4-OMe	55	220	58	46	184	52	26	104	−46
4	4c, R = 4-Cl	20	80	65	19	76	53	24	96	−50
5	5c, R = 4-F	23	92	66	17	68	41	29	116	−61
6	6c, R = 3, 4-F	24	86	54	20	80	40	27	108	−72
7	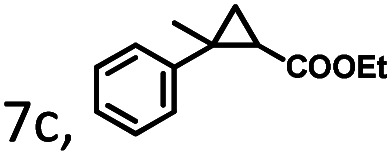	39	156	17	35	90	10	30	120	−3
0.5										
	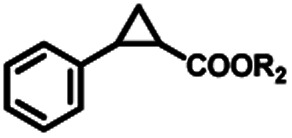									
8	8c, R_2_ = *t*-Bu	39	186	82	35	140	60	18	72	−35
9	9c, R_2_ = CH(i-Pr)_2_	47	188	85	41	164	68	23	92	−35
10	10c, R_2_ = CCH_3_(i-Pr)_2_	41	164	91	39	156	64	27	108	−31
11	11c, R_2_ = CH(Cy)_2_	42	168	36	40	160	25	29	116	−11

a The experiments were carried out with styrene (30 mM), EDA (10 mM), DNA (12.5 μM), and FeTMPyP*n* (*n* = 2, 3, 4) (12.5 μM) in 10 mM potassium phosphate buffer (pH 7.0) under argon at 4 °C, 2 h, unless otherwise specified. Product conversions and ee values are based on the areas of HPLC peaks compared to an internal standard. The diastereomeric ratios of the products ranged from 86 : 14 to 97 : 3. All data are the average of 3 attempts, reproducibility ±5%.

b (*R*,*R*)–(*S*,*S*).

## Conclusions

In conclusion, stereo-divergence of G4 biocatalyst-catalyzed olefin cyclopropanation was achieved *via* cofactor modification. By tuning the *N*-methyl substituent of the porphyrin ligand in the cofactor from the *para*- to the *ortho*-position, the self-assembled G4–Fe-porphyrin biocatalysts are able to switch the enantioselectivity of the reaction from +91% to −72% ee_trans_. CD, NMR, ITC, and other characterization studies reveal that the porphyrin ligand cooperating with the deoxynucleotide residues gives the IPC intermediate a single well-defined configuration and results in a specific enantiopreference. This finding is down to the rational design of DNA-based biocatalysts through cofactor modification, a method which serves as an effective way to regulate the catalytic performance of DNA-based biocatalysts.

## Author contributions

J. Y. Hao conceived of the presented idea. J. Y. Hao, W. H. Miao, and S. M. Lu planned the experiments. J. Y. Hao and W. H. Miao carried out the experiments. J. Y. Hao, W. H. Miao, S. M. Lu, Y. Cheng, and C. Li contributed to the interpretation of the results. J. Y. Hao, S. M. Lu, and C. Li wrote the manuscript. C. Li supervised the project. All authors contributed to the final manuscript.

## Conflicts of interest

There are no conflicts to declare.

## Supplementary Material

SC-012-D1SC00755F-s001
