# Supplementary material for: Controllable stereoinversion in DNA-catalyzed olefin cyclopropanation via cofactor modification
Source: Chem Sci. 2021 May 11;12(22):7918–23. doi: 10.1039/d1sc00755f (PMC8188488; doi:10.1039/d1sc00755f)
Supplement: SC-012-D1SC00755F-s001 [file SC-012-D1SC00755F-s001.pdf]

Electronic Supplementary Material (ESI) for Chemical Science.
This journal is © The Royal Society of Chemistry 2021

Supporting Information

Controllable Stereoinversion in DNA-catalyzed Olefin Cyclopropanation via Cofactor Modification

Jingya Hao,[1, 2] Wenhui Miao,[1, 2] Shengmei Lu,[1] Yu Cheng,[1, 2] Guoqing Jia,[1] Can Li[*, 1]

[1]*State Key Laboratory of Catalysis, Dalian Institute of Chemical Physics, Chinese Academy of Sciences, Zhongshan Road 457, Dalian 116023, China*

[2]*University of Chinese Academy of Sciences, Beijing 101408, China*

Table of Contents

Materials and Methods 3

General Procedures 7

Supplementary Results 10

Synthesis and Characterization 13

HPLC Traces 24

References 46

Materials and Methods

Unless otherwise noted, all chemicals and reagents for chemical reactions were obtained from commercial suppliers (Sigma-Aldrich, Acros, TCI, Frontier scientific) and used without further purification. The DNA sequences were all purchased from Sangon (Shanghai, China). The DNA strand concentrations were determined by measuring the UV absorbance of sample at 260 nm by using the molar extinction coefficient values provided by the manufacturer. Water purified on a Milli-Q A10 water purification system (specific resistance of 18.2 MΩ at 25 ^{o}C) was used for all experiments.

High Performance Liquid Chromatography (HPLC). The enantioselectivity was determined by Agilent HPLC 1260 analysis using Daicel chiralcel OJH column and Daicel CHIRALPAK-IJ column with a UV-detector by using ethanol, isopropanol and n-hexane as eluents at 25 °C.

Circular Dichroism (CD) Spectroscopy. All CD spectra were recorded on a dual beam DSM 1000 CD spectrophotometer (Olis, Bogart, GA) with a 10 mm or 1.5 mm path-length quartz cell. Each measurement was recorded from 220 to 400 nm at 20 ºC under N_2 purge. The scan rate was 0.5 nm per second. The average scan for each sample was subtracted by a background CD spectrum of corresponding buffer solution.

UV Melting Experiment. UV melting experiments were carried out on Shimadzu 2450

spectrophotometer (Shimadzu, Japan) equipped with a Peltier temperature control accessory. A sealed quartz cell with a path length of 1.0 cm was used. The UV melting curves of the G-quadruplexes and G4-based biocatalysts were monitored by UV absorption at 295 nm with a heating rate of 0.5 °C/min. Data were analyzed by using Origin 8 software. The melting temperatures (T_m) can be obtained from the best sigmoidal curve fit of the melting profile.

UV-Vis Absorption Titration Experiments. Absorption spectra were measured on Shimadzu 2600 spectrophotometer (Shimadzu, Japan) with a 1 cm path-length quarter cell. UV-vis absorption titrations were carried out by the stepwise addition of G-quadruplex solution to a cell containing FeTMPyPn (n = 4, 3, 2). Absorption spectra were recorded in the range of 300-550 nm at room temperature. The titration was terminated when the wavelength and intensity of the Soret band for FeTMPyPn did not change any more upon three successive additions of G-quadruplexes.

Nuclear Magnetic Resonance (NMR) Titration. NMR titration experiments were performed on Bruker-700MHz NMR instrument in potassium phosphate buffer (10mM, pH 7.0), containing 5% D_2O. The strand concentration of NMR samples was 0.25 mM. Before experiment the samples were heated at 95 °C for 3 min and annealed to room temperature.

Isothermal Titration Calorimetry (ITC).[1] ITC measurements were carried out at 25

°C using a MicroCal TM ITC 200 titration calorimeter (MicroCal, GE). Experiments were performed in potassium phosphate buffer (10mM, pH 7.0). The reference cell in the ITC was filled with ultrapure water (18.2 MΩ). A pre-folded G-quadruplex DNA was loaded into the calorimeter cell. Then the syringe was loaded with FeTMPyPn (n = 4, 3, 2) (1.5 mM) in corresponding buffers. Following the auto-equilibration and an initial 60 s delay, the FeTMPyPn titrant divided into 25 injections was added into the cell with 250s injection intervals. The stir rate was 1000 rpm. All data were recorded with the GE Instruments software provided. Calorimetric data were further analyzed according to relevant model using MicroCal ORIGIN software and MATLAB. Data analysis gives ΔH (binding enthalpy change, k_{cal}/mol), K_a (binding constant, M^{-1}), and n (number of bound FeTMPyPn cofactor) whereas the change in Gibbs energy and the entropic contribution were determined by the relationships $\Delta G = -RT\ln K_a$ and $\Delta G = \Delta H - T\Delta S$, respectively.

Fluorescence quench titration assay.[2] Fluorescently labelled oligonucleotide was dissolved in assay buffer (potassium phosphate buffer 10 mM, pH 7.0), which is in agreement with the catalytic buffer. The resultant strand concentration of oligonucleotide was 100 nM. FeTMPyPn (n = 4, 3, 2) was prepared at a concentration of 5 mM in water and diluted to an appropriate concentration before titration. The fluorescence experiments were recorded on a FLS920 fluorescence spectrometer (Edinburgh) with a 1 cm path length quartz cuvette at 20 °C. Fluorescence intensity of FAM-labeled DNA was recorded after the addition of FeTMPyPn. Interval time

between two titration points was 10-15 minutes in order to reach the binding equilibrium. Each quench titration assay was conducted in triplicate. If not stated otherwise, the titration curve was fitting as one site specific binding by using software Origin 8.1.

General Procedures

Typical procedure for cyclopropanation bioconversions under anaerobic conditions. Reactions (1 mL) were conducted in 10 mL Schlenk tubes (Synthware Glass, Beijing). G4-based biocatalysts were added to the tube with a small stir bar in phosphate buffer (10 mM, pH = 7.0) and a solution of the reductant ($Na_2S_2O_4$, or NADPH) were combined in tube and degassed by bubbling argon through the solution for 5 min. The headspace of tube was made anaerobic by flushing argon over the solution (with no bubbling). A styrene solution in DMSO (20 μL, typically 1.5 M) was added to the reaction vial via a glass syringe, and left to stir for about 30s. An EDA solution in DMSO was then added (20 μL, 0.5 M) and the reaction was left to stir for appropriate time. The final concentrations of the reagents were typically: 30 mM styrene, 10 mM EDA, 5 mM $Na_2S_2O_4$ or 0.5 mM NADPH, 12.5 μM mA9A-FeTMPyPn (n = 4, 3, 2). After 2 hours of reaction, the product was extracted with ethyl acetate (3 × 2 mL). The organic layer was washed with brine (1 × 5 mL). After a short flash chromatography containing anhydrous Na_2SO_4 and the evaporation of solvent, the crude product was analyzed by HPLC, using 2-methylanisole as internal standard.

Synthesis of cyclopropane products.[3] Under inert gas conditions, $[Cu(MeCN)_4]PF_6$ (52 mg) was dissolved in 20 mL anhydrous dichloromethane. Under stirring, olefin (140 mmol) was added to the solution and stirred for further 90 min at room temperature. Subsequently, a solution of ethyl diazoacetate or butyldiazoacetate (14 mmol) in 20 mL anhydrous dichloromethane was dropped to the solution over 4 hours.

The reaction mixture was allowed to stirrer at room temperature overnight. The pure product was obtained after flash chromatography.

Synthesis of cofactor FeTMPyPn.[4]

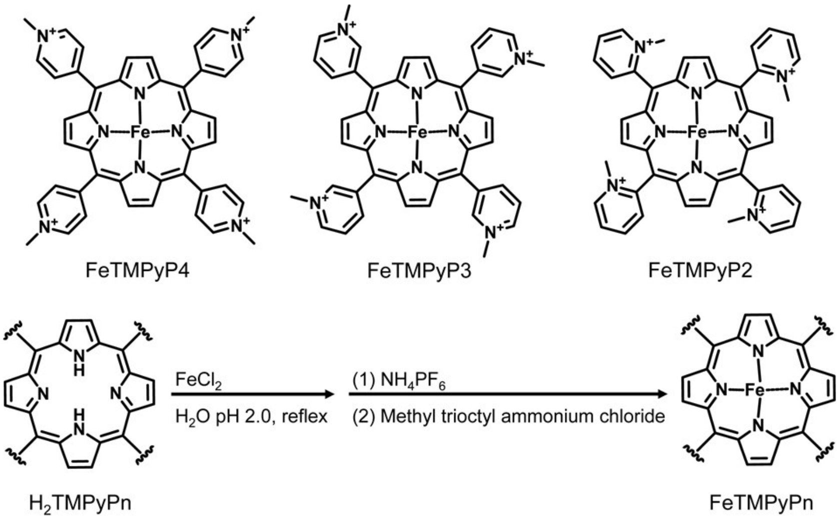

The pH of an aqueous solution of H_2TMPyP (0.15 mmol) in 60 ml water was adjusted to 2 (with 1 M HCl), and a 40-fold molar excess $FeCl_2.4H_2O$ was added and the solution was stirred and heated under reflux. The course of the metalation was followed by the decrease of the fluorescence of the metal-free porphyrin using UV light at 356 nm. The metalation was completed in 24 hours. The solution was filtered through a filter paper. The Fe porphyrin was precipitated as the PF_6^- salt with a saturated aqueous solution of NH_4PF_6 (2 ml). The precipitate was thoroughly washed with diethyl ether (5 × 5 mL). The dried precipitate was then dissolved in acetone (the smallest possible amount) and precipitated as the chloride salt with saturated acetone solution of methyl-tri-noctylammonium chloride (2 mL). The precipitate was washed with acetone and dissolved in the smallest possible amount of water. The whole precipitation procedure was repeated once again to ensure high purity.

Synthesis of diazo esters.[5]

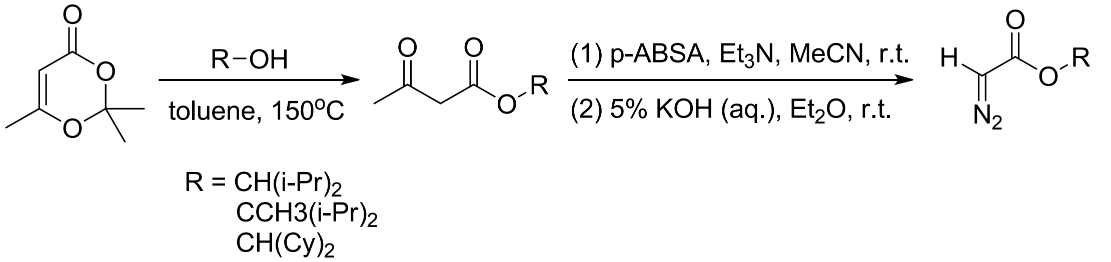

A solution of R-OH (50 mmol) and 2,2,6-trimethyl-4H-1,3-dioxin-4-one (50 mmol) in 10 mL of toluene was placed in a 50-ml flask. The flask was immersed in an oil bath that had been preheated to 150 °C, and the solution was vigorously stirred. The evolution of acetone became apparent within several minutes, heating was continued for a total of 6 hours. The reaction was cooled, and then the toluene was removed, and the product was distilled. To the solution of first step product (10 mmol) in acetonitrile (12ml) was added Et_3N (13 mmol). The reaction mixture was cooled in an ice bath and a solution of p-ABSA (11 mmol) in acetonitrile (12 ml) was added slowly. The reaction mixture was allowed to warm to r.t. After stirring for 10h, solvent was removed under reduced pressure. The residue was dissolved in ether (60 ml) and washed with 5% aqueous KOH solution. To a solution of the crude product in ethyl ether was added 5% KOH (50 ml), and the reaction mixture was stirred for 1h. The organic phase was separated, dried over Na_2SO_4 , and concentrated under reduced pressure. Purification by vacuum distillation provided the desired diazo esters as yellow liquid.

Supplementary Results

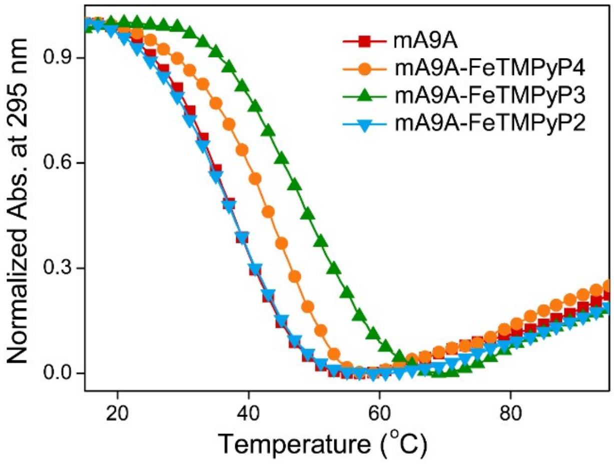

Figure S1. UV-melting spectra of mA9A and mA9A-FeTMPyPn (n = 4, 3, 2).

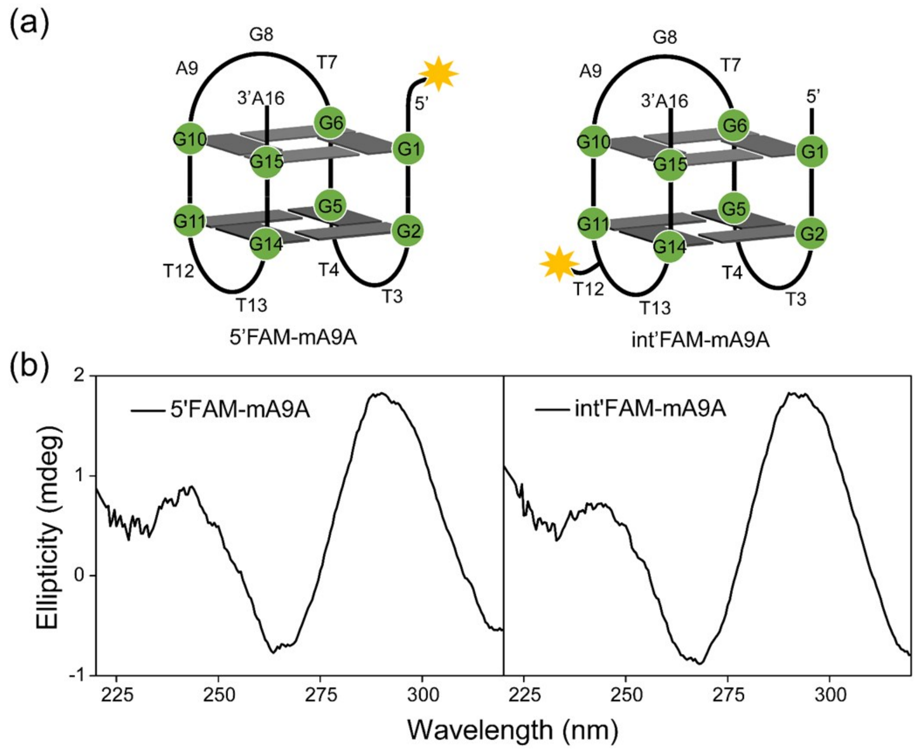

Figure S2. (a) The diagrams of site-specific FAM labelled mA9A G-quadruplex. (b) CD spectra of FAM labelled mA9A (FAM labelled G4 strand concentration 15 μM, potassium phosphate buffer 10 mM, pH 7.0).

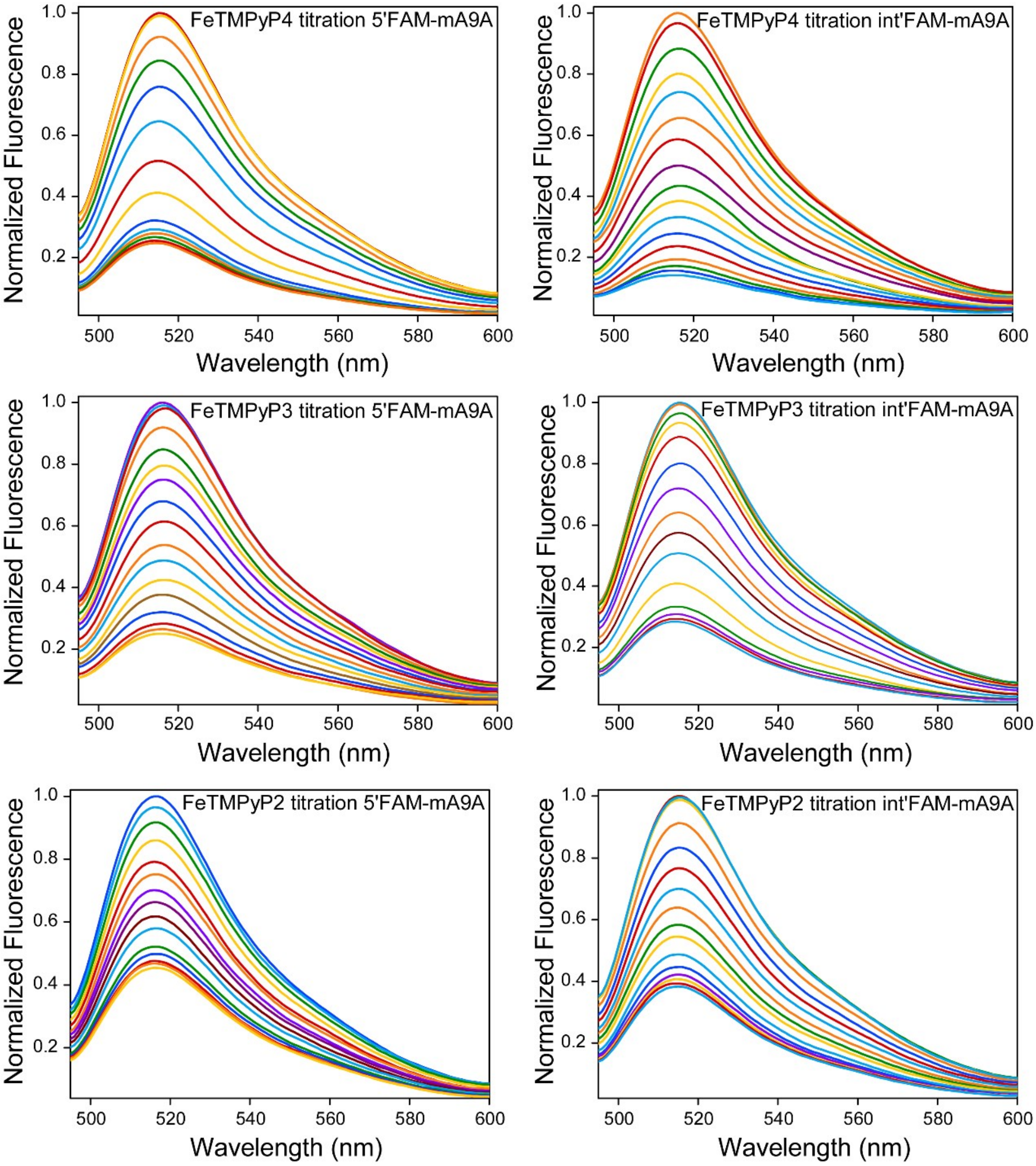

Figure S3. FeTMPyPn (n = 4, 3, 2)-dose-responsive FAM-mA9A emission spectra.

Synthesis and Characterization

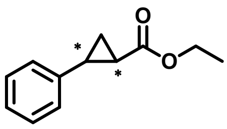

(1RS, 2RS)-Ethyl 2-phenylcyclopropane-1-carboxylate.[4] Prepared using general procedure, starting from styrene. Purified by column chromatography (SiO_2, EtOAc: pentane = 1: 10), to afford the product as a white solid.

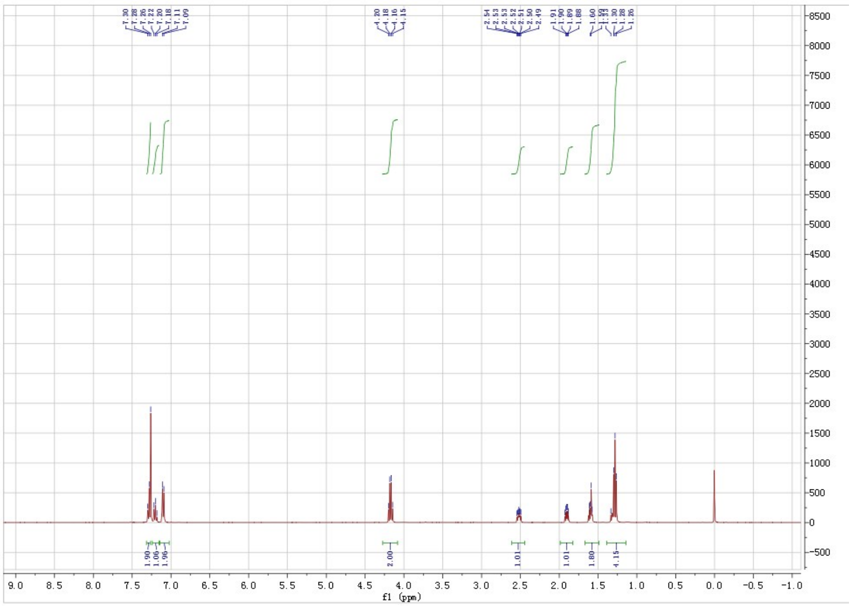

HPLC analysis condition: Daicel Chiralcel-OJH, n-hexane, flow rate 1 mL/min, λ = 225 nm. 2-methylanisole as internal standard.

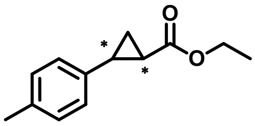

(1RS, 2RS)-Ethyl 2-(4-methylphenyl) cyclopropane-1-carboxylate.[6] Prepared using general procedure, starting from 4-methylstyrene. Purified by column chromatography (SiO_2, EtOAc: pentane = 1: 10), to afford the product as a white solid.

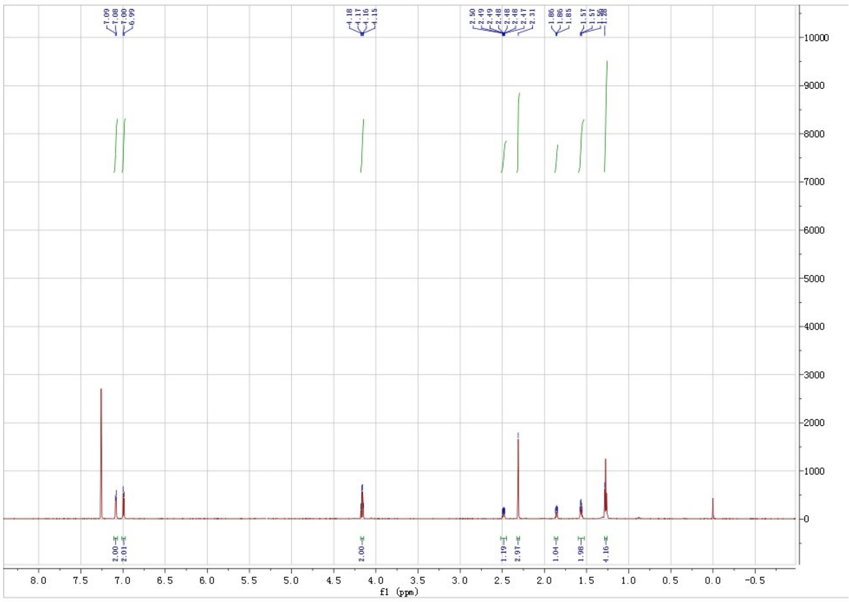

HPLC analysis condition: Daicel Chiralcel-OJH, n-hexane, flow rate 1 mL/min, λ = 225 nm. 2-methylanisole as internal standard.

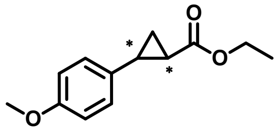

(1RS, 2RS)-Ethyl 2-(4-methoxyphenyl) cyclopropane-1-carboxylate.[4] Prepared using general procedure, starting from 4-methoxystyrene. Purified by column chromatography (SiO_2, EtOAc: pentane = 1: 20), to afford the product as liquid.

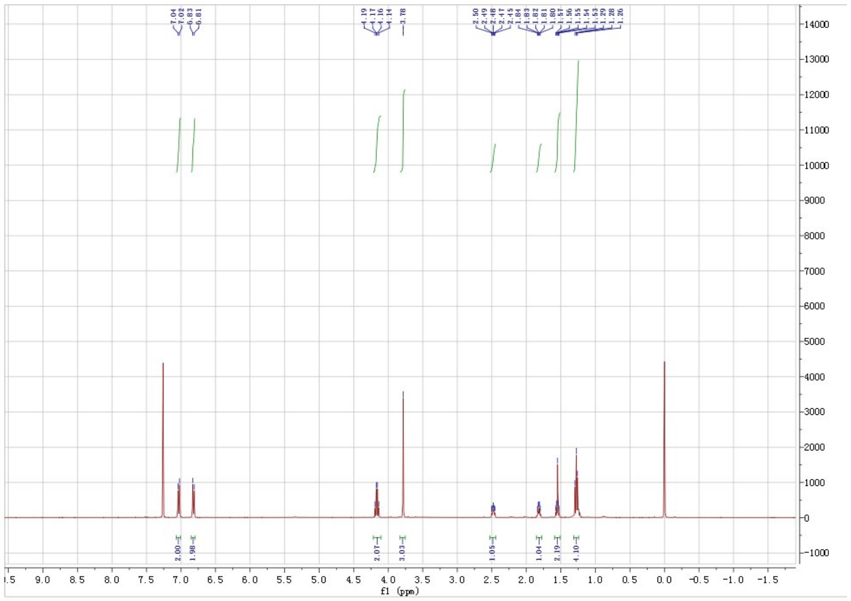

HPLC analysis condition: Daicel Chiralcel-OJH, n-hexane: ethanol = 98: 2, flow rate 0.8 mL/min, λ = 225 nm. 2-methylanisole as internal standard.

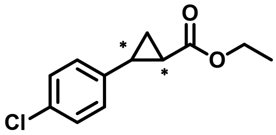

(1RS, 2RS)-Ethyl 2-(4-chlorophenyl) cyclopropane-1-carboxylate.[4] Prepared using general procedure, starting from 4-chlorostyrene. Purified by column chromatography (SiO_2, EtOAc: pentane = 1: 50), to afford the product as a white solid.

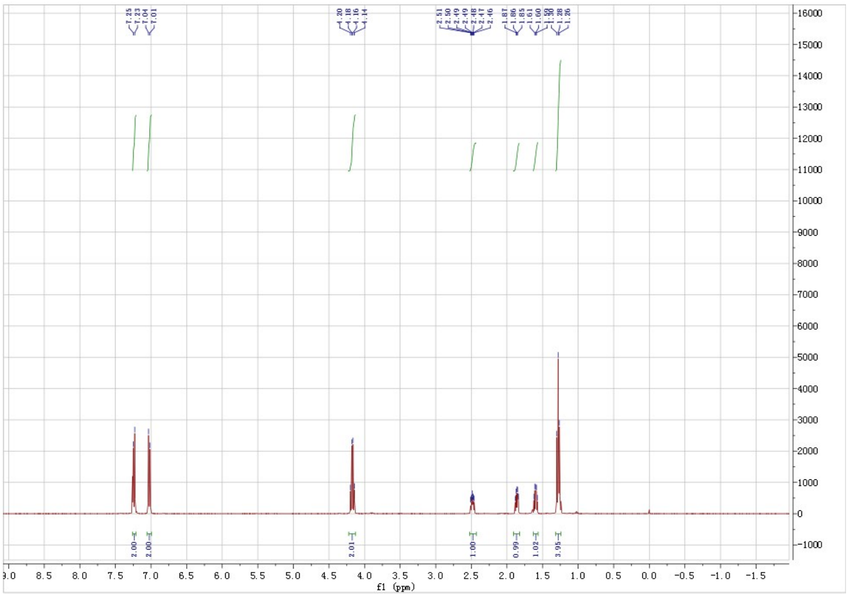

HPLC analysis condition: Daicel Chiralcel-OJH, n-hexane: ethanol = 98: 2, flow rate 0.5 mL/min, λ = 225 nm. 2-methylanisole as internal standard.

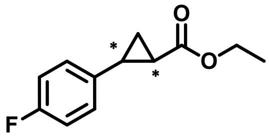

(1RS, 2RS)-Ethyl 2-(4-fluorophenyl) cyclopropane-1-carboxylate.[7] Prepared using general procedure, starting from 4-fluorostyrene. Purified by column chromatography (SiO_2, EtOAc: pentane = 1: 50), to afford the product as a white solid.

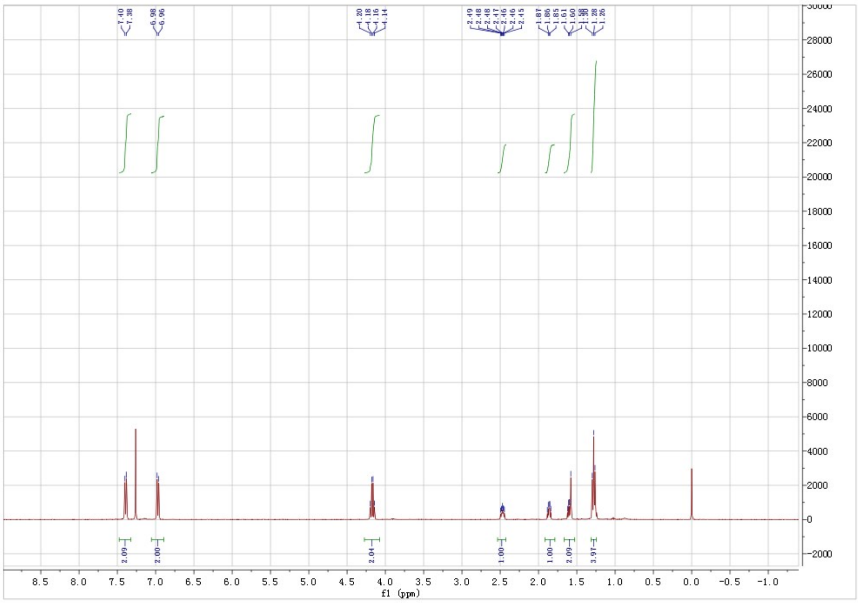

HPLC analysis condition: Daicel Chiralcel-OJH, n-hexane: ethanol = 98: 2, flow rate 0.5 mL/min, λ = 225 nm. 2-methylanisole as internal standard.

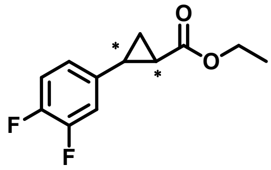

(1RS, 2RS)-Ethyl 2-(3,4-difluorophenyl) cyclopropane-1-carboxylate.[8] Prepared using general procedure, starting from 3,4-difluorostyrene. Purified by column chromatography (SiO_2, EtOAc: pentane = 1: 50), to afford the product as pale yellow liquid.

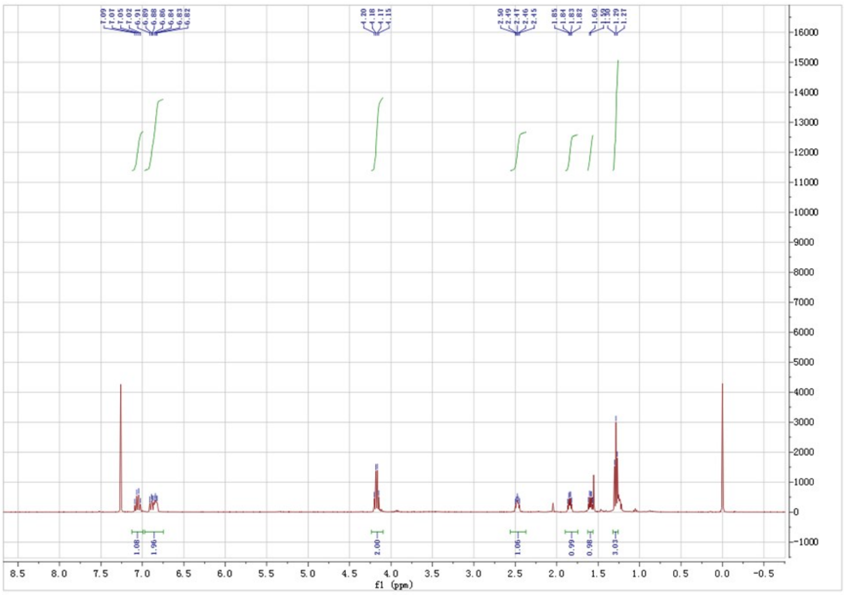

HPLC analysis condition: Daicel CHIRALPAK-IJ, n-hexane: isopropanol = 98: 2, flow rate 0.3 mL/min, λ = 235 nm. 2-methylanisole as internal standard.

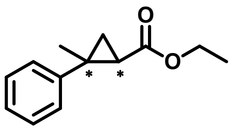

(1RS, 2RS)-Ethyl 2-methyl-2-phenylcyclopropane-1-carboxylate.[6]

Prepared using general procedure, starting from 2-phenyl-1-propene. Purified by column chromatography (SiO_2, EtOAc: pentane = 1: 10), to afford the product as a white solid.

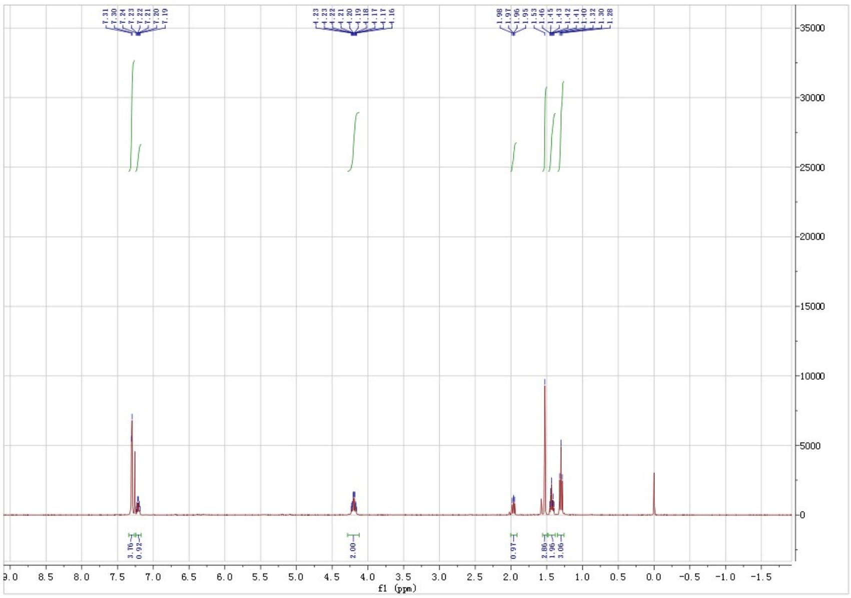

HPLC analysis condition: Daicel Chiralcel-OJH, n-hexane, flow rate 1 mL/min, λ = 225 nm. Thioanisole as internal standard.

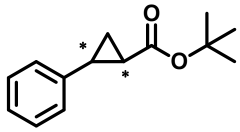

(1RS, 2RS)-tert-Butyl 2-phenylcyclopropane-1-carboxylate.[6] Prepared using general procedure, starting from styrene and *t*-BuDA. Purified by column chromatography (SiO_2, EtOAc: pentane = 1: 30), to afford the product as pale yellow liquid.

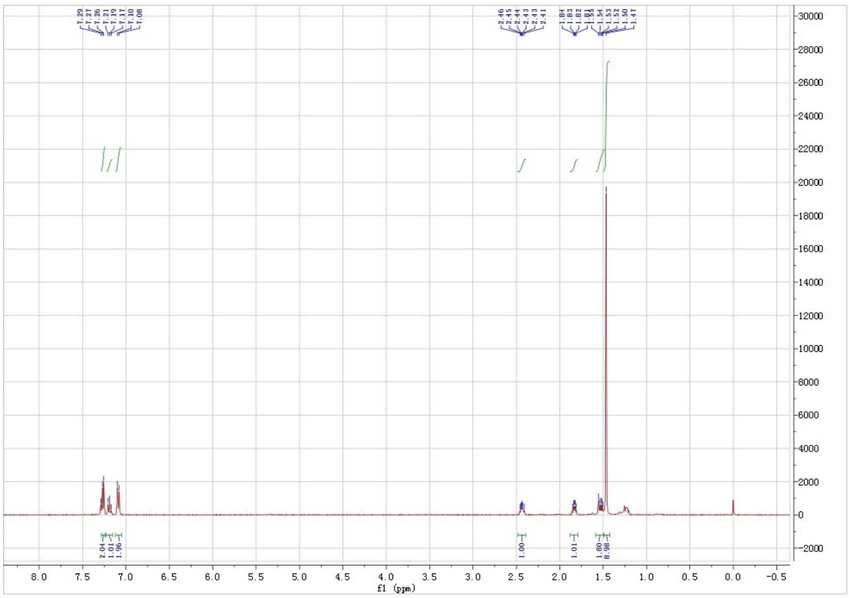

HPLC analysis condition: Daicel CHIRALPAK-IJ, n-hexane, flow rate 1 mL/min, λ = 225 nm. Thioanisole as internal standard.

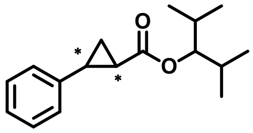

(1RS, 2RS)- 2-methyl-1-(1-methylethyl)propyl 2-phenylcyclopropane-1-carboxylate.[9] Prepared using general procedure, starting from 2-Methyl-1-(1-methylethyl)propyl 2-diazoacetate. Purified by column chromatography (SiO_2, EtOAc: pentane = 1: 30), to afford the product as pale yellow liquid.

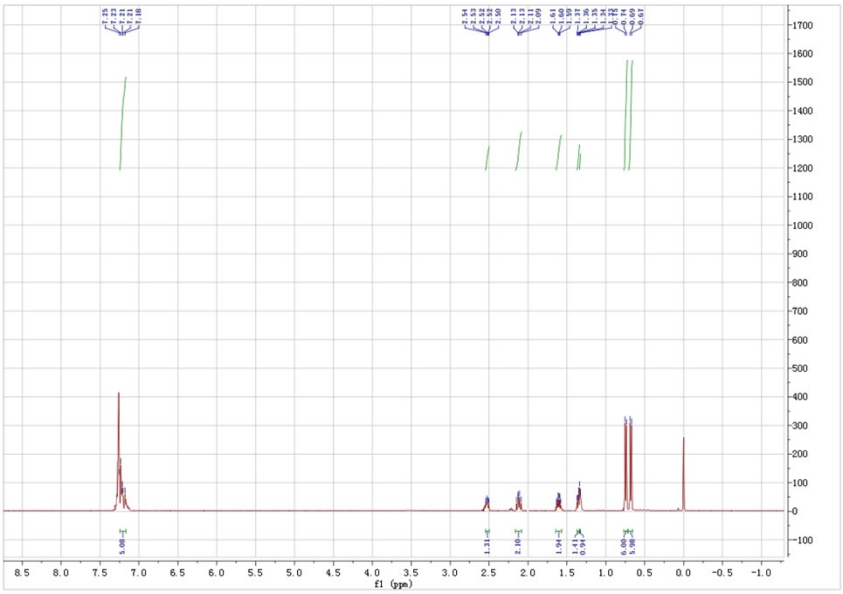

HPLC analysis condition: Daicel CHIRALPAK-IJ, n-hexane, flow rate 0.8 mL/min, λ = 225 nm. 4-Methoxystrene as internal standard.

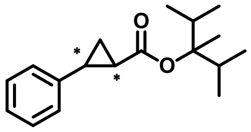

(1RS, 2RS)- 1,2-dimethyl-1-(1-methylethyl)propyl 2-phenylcyclopropane-1-carboxylate.[10] Prepared using general procedure, starting from 2,3,4-Trimethyl-3-pentyl diazoacetate. Purified by column chromatography (SiO_2, EtOAc: pentane = 1: 50), to afford the product as pale yellow liquid.

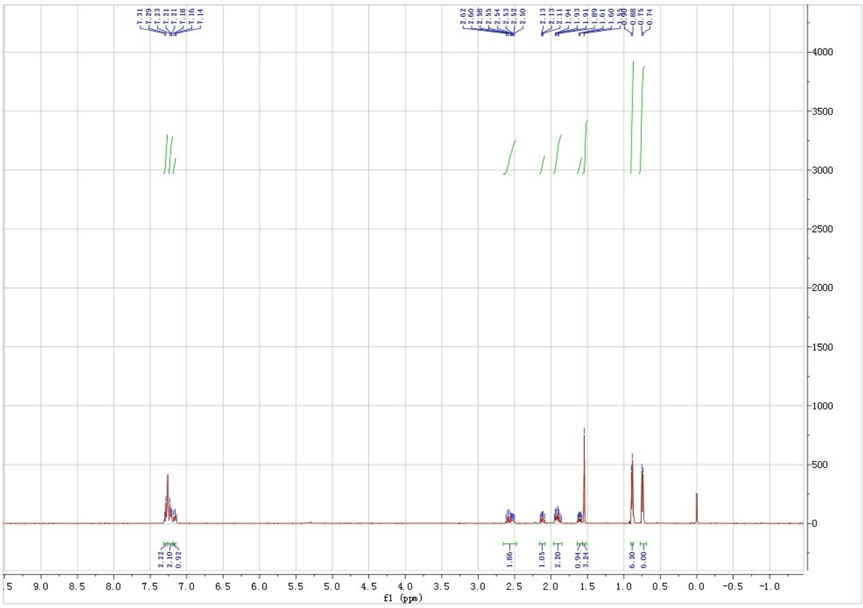

HPLC analysis condition: Daicel CHIRALPAK-IJ, n-hexane, flow rate 0.5 mL/min, λ = 225 nm. 4-Methoxystrene as internal standard.

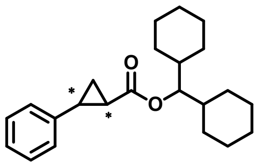

(1RS, 2RS)- dicyclohexylmethyl 2-phenylcyclopropane-1-carboxylate.[11] Prepared using general procedure, starting from 2,3,4-Trimethyl-3-pentyl diazoacetate. Purified by column chromatography (SiO_2, EtOAc: pentane = 1: 30), to afford the product as pale yellow liquid.

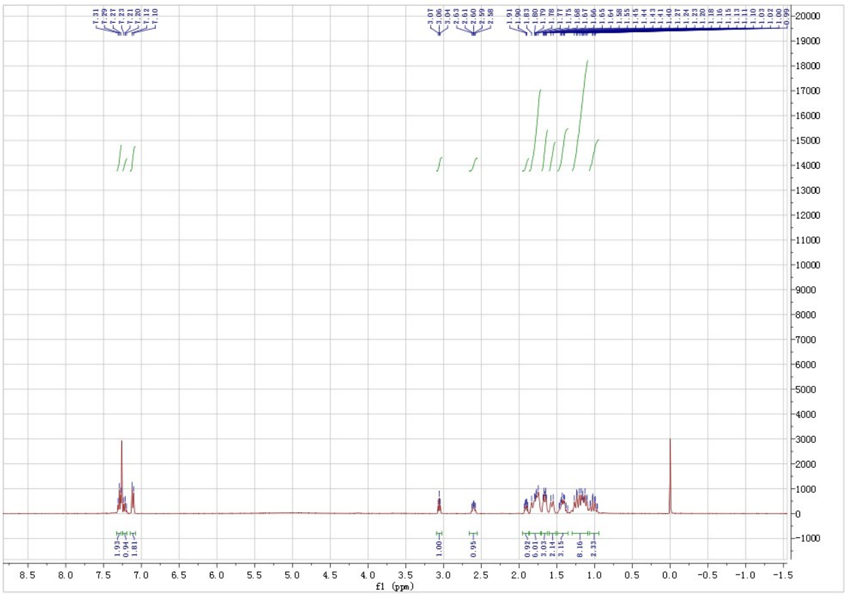

HPLC analysis condition: Daicel Chiralcel-OJH, n-hexane, flow rate 0.6 mL/min, λ = 235 nm. Thioanisole as internal standard.

HPLC Traces of Products

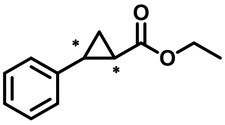

(1) Racemic *trans* product catalyzed by FeTMPyP4

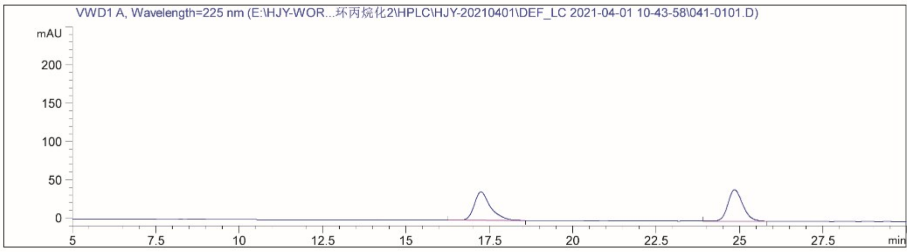

Signal 1: VWD1 A, Wavelength=225 nm

Peak #	RetTime [min]	Type	Width [min]	Area [mAU*s]	Height [mAU]	Area %
1	17.246	BB	0.5033	1276.79822	37.38312	50.1381
2	24.850	VB	0.4749	1269.76685	41.00116	49.8619
Totals:				2546.56506	78.38428	

(2) Racemic *trans* product catalyzed by FeTMPyP3

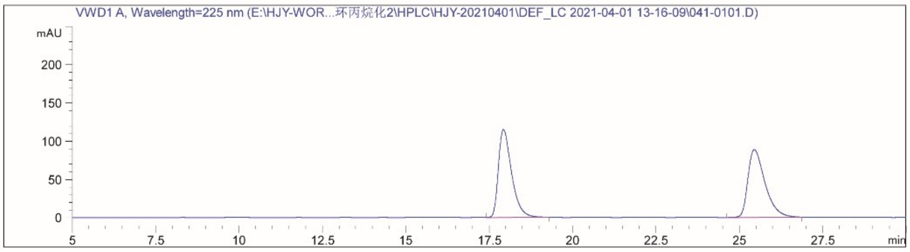

Signal 1: VWD1 A, Wavelength=225 nm

Peak #	RetTime [min]	Type	Width [min]	Area [mAU*s]	Height [mAU]	Area %
1	17.929	VB	0.4202	3165.36792	114.99096	49.9849
2	25.449	BB	0.5448	3167.27930	88.68572	50.0151
Totals:				6332.64722	203.67668	

(3) Racemic *trans* product catalyzed by FeTMPyP2

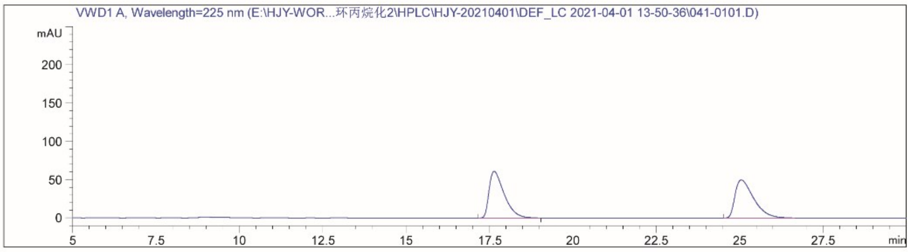

Signal 1: VWD1 A, Wavelength=225 nm

Peak #	RetTime [min]	Type	Width [min]	Area [mAU*s]	Height [mAU]	Area %
1	17.639	BB	0.4778	1929.94141	61.08487	49.8039
2	25.047	BB	0.5935	1945.14063	49.85826	50.1961
Totals:				3875.08203	110.94313	

(4) *Trans* product from the cyclopropanation catalyzed by mA9A-FeTMPyP2

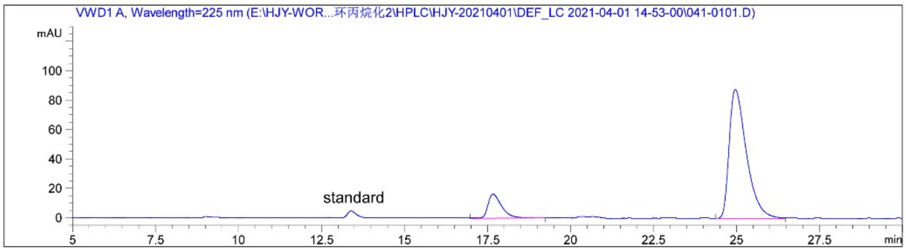

Signal 1: VWD1 A, Wavelength=225 nm

Peak #	RetTime [min]	Type	Width [min]	Area [mAU*s]	Height [mAU]	Area %
1	17.670	MM	0.4822	472.53473	16.33153	12.9650
2	24.968	VB	0.5528	3172.17334	87.74774	87.0350
Totals:				3644.70807	104.07927	

(5) *Trans* product from the cyclopropanation catalyzed by mA9A-FeTMPyP3

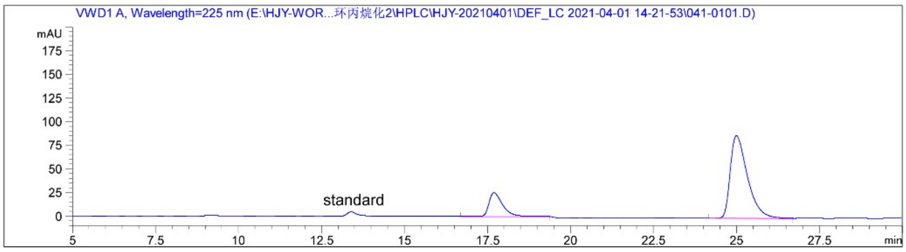

Signal 1: VWD1 A, Wavelength=225 nm

Peak #	RetTime [min]	Type	Width [min]	Area [mAU*s]	Height [mAU]	Area %
1	17.698	MM	0.4853	734.51807	25.22478	18.8650
2	25.001	BB	0.5467	3159.02954	87.44763	81.1350
Totals:				3893.54761	112.67242	

(6) *Trans* product from the cyclopropanation catalyzed by mA9A-FeTMPyP2

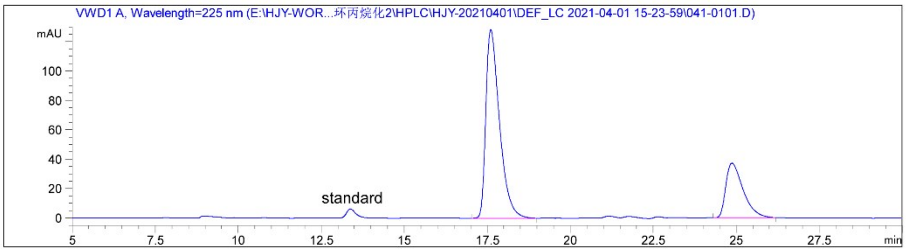

Signal 1: VWD1 A, Wavelength=225 nm

Peak #	RetTime [min]	Type	Width [min]	Area [mAU*s]	Height [mAU]	Area %
1	17.606	BB	0.4342	3670.93481	128.31760	73.2178
2	24.872	BB	0.5489	1342.78650	37.10139	26.7822
Totals:				5013.72131	165.41899	

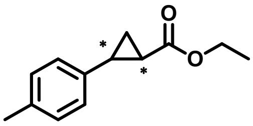

(1) Racemic *trans* product catalyzed by FeTMPyP4

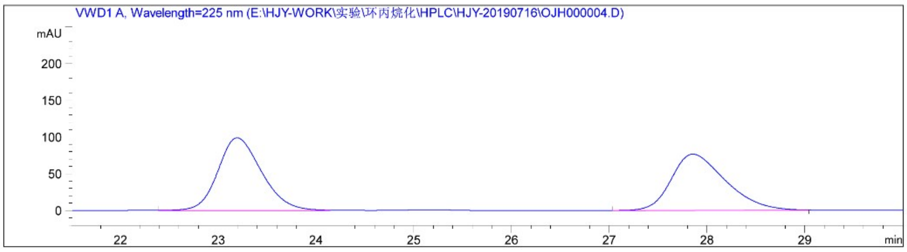

Signal 1: VWD1 A, Wavelength=225 nm

Peak #	RetTime [min]	Type	Width [min]	Area [mAU*s]	Height [mAU]	Area %
1	22.490	MF	0.4560	2707.14380	98.95305	50.7884
2	26.602	MF	0.5702	2623.10059	76.67128	49.2116
Totals:				5330.24438	175.62433	

(2) Racemic *trans* product catalyzed by FeTMPyP3

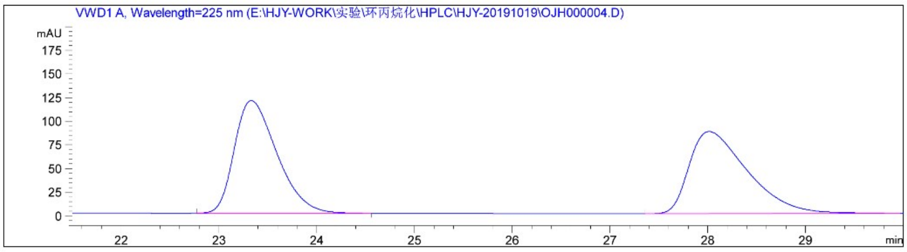

Signal 1: VWD1 A, Wavelength=225 nm

Peak #	RetTime [min]	Type	Width [min]	Area [mAU*s]	Height [mAU]	Area %
1	23.333	BB	0.4669	3607.62500	119.15497	50.0601
2	28.016	BB	0.6321	3598.96021	86.85562	49.9399
Totals:				7206.58521	206.01059	

(3) Racemic *trans* product catalyzed by FeTMPyP2

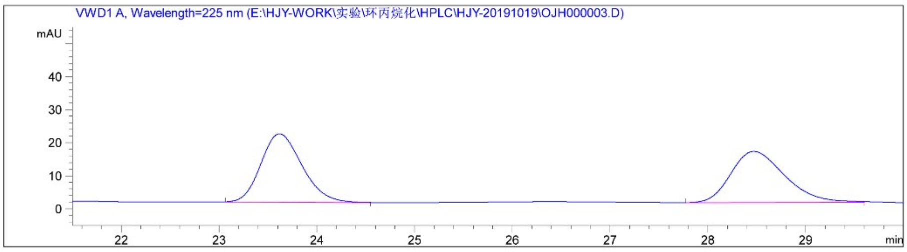

Signal 1: VWD1 A, Wavelength=225 nm

Peak #	RetTime [min]	Type	Width [min]	Area [mAU*s]	Height [mAU]	Area %
1	23.620	BB	0.4530	608.94958	20.75915	50.6158
2	28.471	BB	0.5904	594.13354	15.48688	49.3842
Totals:				1203.08313	36.24603	

(4) *Trans* product from the cyclopropanation catalyzed by mA9A-FeTMPyP4

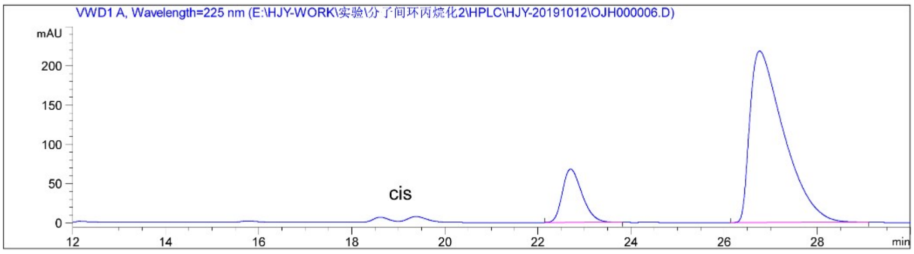

Signal 1: VWD1 A, Wavelength=225 nm

Peak #	RetTime [min]	Type	Width [min]	Area [mAU*s]	Height [mAU]	Area %
1	22.724	MF	0.4883	1773.17981	60.51929	15.2097
2	26.848	BB	0.7475	9885.05078	197.88264	84.7903
Totals:				1.16582e4	258.40193	

(5) *Trans* product from the cyclopropanation catalyzed by mA9A-FeTMPyP3

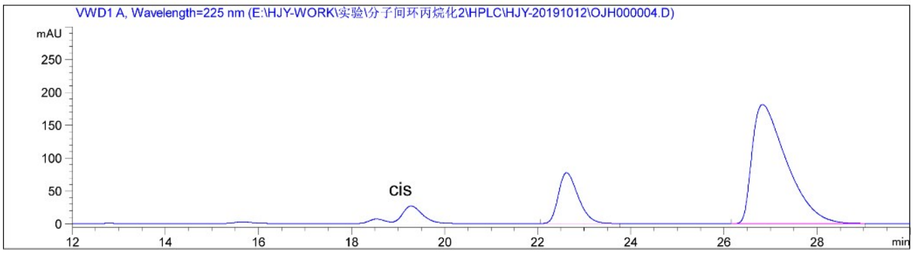

Signal 1: VWD1 A, Wavelength=225 nm

Peak #	RetTime [min]	Type	Width [min]	Area [mAU*s]	Height [mAU]	Area %
1	22.622	BB	0.4584	2293.74512	77.30296	19.6823
2	26.834	BB	0.7756	9360.07715	181.36884	80.3177
Totals:				1.16538e4	258.67180	

(6) *Trans* product from the cyclopropanation catalyzed by mA9A-FeTMPyP2

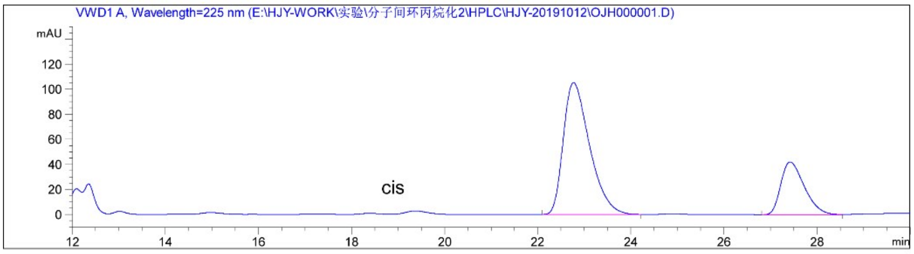

Signal 1: VWD1 A, Wavelength=225 nm

Peak #	RetTime [min]	Type	Width [min]	Area [mAU*s]	Height [mAU]	Area %
1	22.772	BB	0.6007	4132.17041	105.30273	73.5773
2	27.425	BB	0.5446	1483.92273	42.17602	26.4227
Totals:				5616.09314	147.47876	

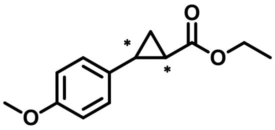

(1) Racemic *trans* product catalyzed by FeTMPyP4

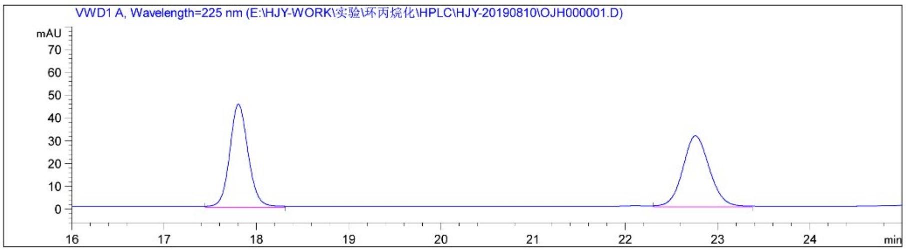

Signal 1: VWD1 A, Wavelength=225 nm

Peak #	RetTime [min]	Type	Width [min]	Area [mAU*s]	Height [mAU]	Area %
1	17.329	BB	0.3045	930.77545	46.88914	50.1834
2	24.542	BB	0.4425	923.97205	32.21429	49.8166
Totals:				1854.74750	79.10344	

(2) Racemic *trans* product catalyzed by FeTMPyP3

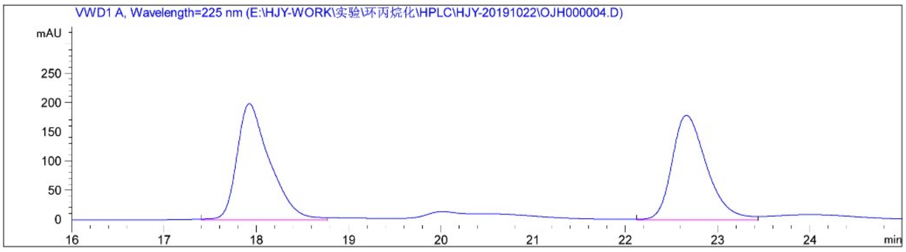

Signal 1: VWD1 A, Wavelength=225 nm

Peak #	RetTime [min]	Type	Width [min]	Area [mAU*s]	Height [mAU]	Area %
1	17.924	FM	0.4126	4927.31396	199.02786	51.1998
2	22.663	BV	0.3989	4696.38379	179.25935	48.8002
Totals:				9623.69775	378.28722	

(3) Racemic *trans* product catalyzed by FeTMPyP2

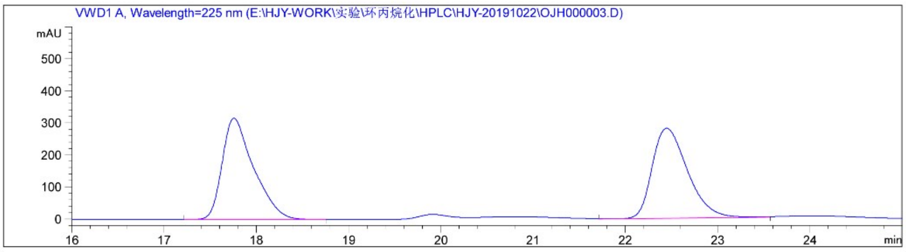

Signal 1: VWD1 A, Wavelength=225 nm

Peak #	RetTime [min]	Type	Width [min]	Area [mAU*s]	Height [mAU]	Area %
1	17.758	BB	0.3457	7474.04639	316.19641	49.9475
2	22.448	MM	0.4440	7489.76221	281.14175	50.0525
Totals:				1.49638e4	597.33817	

(4) *Trans* product from the cyclopropanation catalyzed by mA9A-FeTMPyP4

Additional Info : Peak(s) manually integrated

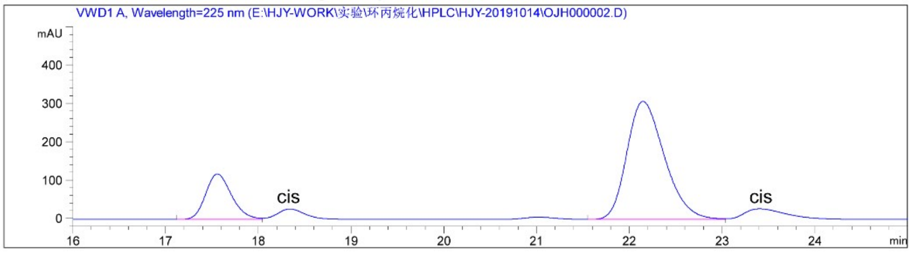

Signal 1: VWD1 A, Wavelength=225 nm

Peak #	RetTime [min]	Type	Width [min]	Area [mAU*s]	Height [mAU]	Area %
1	17.560	BV	0.3003	2313.20581	118.69658	21.0114
2	22.148	VV	0.4373	8696.06348	307.99966	78.9886
Totals:				1.10093e4	426.69624	

(5) *Trans* product from the cyclopropanation catalyzed by mA9A-FeTMPyP3

Additional Info : Peak(s) manually integrated

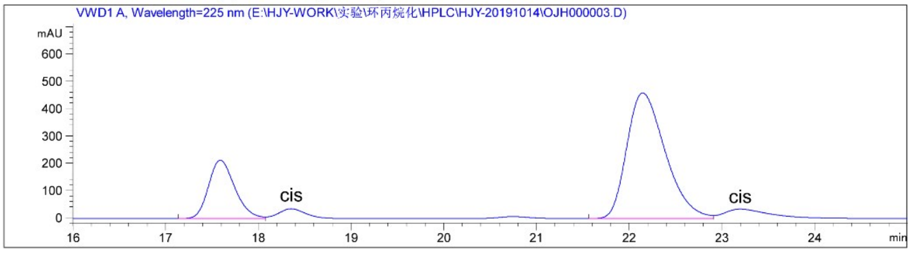

Signal 1: VWD1 A, Wavelength=225 nm

Peak #	RetTime [min]	Type	Width [min]	Area [mAU*s]	Height [mAU]	Area %
1	17.256	BV	0.2975	4117.81885	213.84589	24.0873
2	21.711	BV	0.4339	1.29776e4	460.30869	75.9127
Totals:				1.70954e4	674.15457	

(6) *Trans* product from the cyclopropanation catalyzed by mA9A-FeTMPyP2

Additional Info : Peak(s) manually integrated

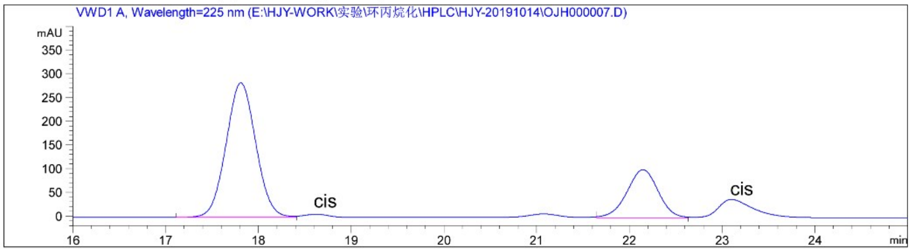

Signal 1: VWD1 A, Wavelength=225 nm

Peak #	RetTime [min]	Type	Width [min]	Area [mAU*s]	Height [mAU]	Area %
1	16.950	BV	0.3482	6344.62109	283.86646	73.0080
2	21.284	FM	0.3869	2345.68286	101.04584	26.9920
Totals:				8690.30396	384.91229	

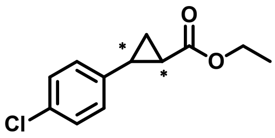

(1) Racemic *trans* product catalyzed by FeTMPyP4

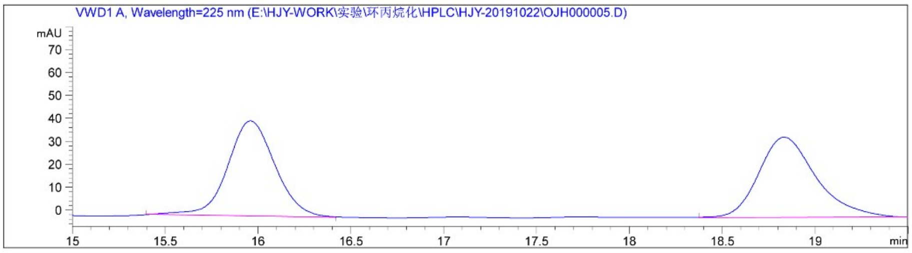

Signal 1: VWD1 A, Wavelength=225 nm

Peak #	RetTime [min]	Type	Width [min]	Area [mAU*s]	Height [mAU]	Area %
1	15.960	MM	0.2946	731.09607	41.35529	49.4399
2	18.834	FM	0.3571	747.66187	34.89382	50.5601

Totals: 1478.75793 76.24911

(2) Racemic *trans* product catalyzed by FeTMPyP3

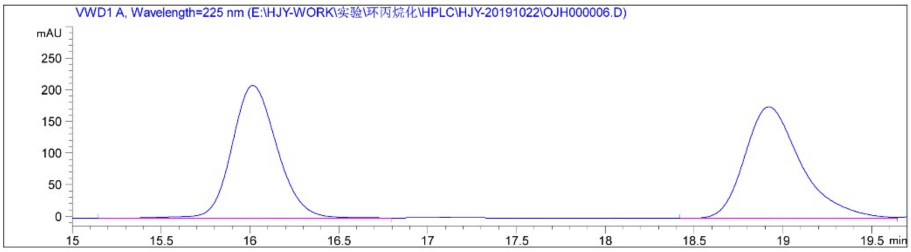

Signal 1: VWD1 A, Wavelength=225 nm

Peak #	RetTime [min]	Type	Width [min]	Area [mAU*s]	Height [mAU]	Area %
1	16.017	VB	0.2739	3718.03223	209.71431	49.0235
2	18.920	VV	0.3337	3866.15039	175.87430	50.9765

Totals: 7584.18262 385.58861

(3) Racemic *trans* product catalyzed by FeTMPyP2

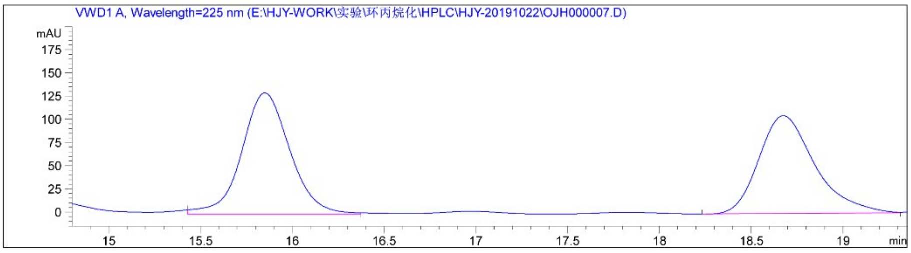

Signal 1: VWD1 A, Wavelength=225 nm

Peak #	RetTime [min]	Type	Width [min]	Area [mAU*s]	Height [mAU]	Area %
1	15.851	MF	0.3030	2373.68726	130.57697	51.4237
2	18.676	VV	0.3235	2242.25073	105.60941	48.5763

Totals: 4615.93799 236.18637

(4) *Trans* product from the cyclopropanation catalyzed by mA9A-FeTMPyP4

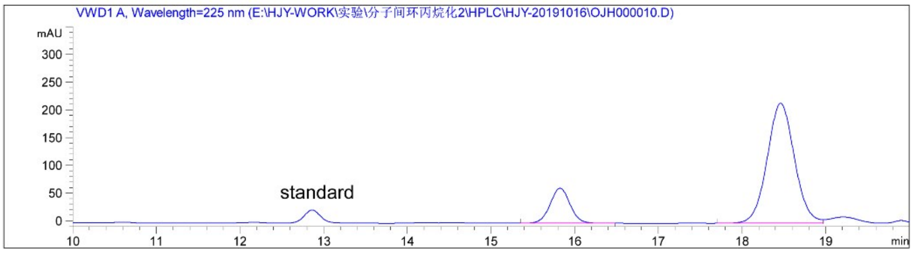

Signal 1: VWD1 A, Wavelength=225 nm

Peak #	RetTime [min]	Type	Width [min]	Area [mAU*s]	Height [mAU]	Area %
1	15.826	BB	0.2557	1045.16516	63.17922	17.5529
2	18.462	BV	0.3497	4909.22412	216.04509	82.4471
Totals:				5954.38928	279.22431	

(5) *Trans* product from the cyclopropanation catalyzed by mA9A-FeTMPyP3

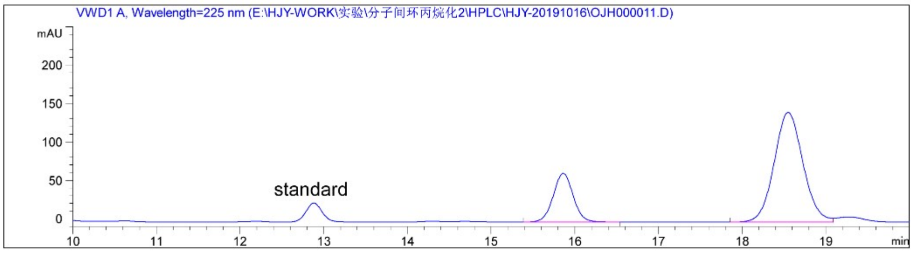

Signal 1: VWD1 A, Wavelength=225 nm

Peak #	RetTime [min]	Type	Width [min]	Area [mAU*s]	Height [mAU]	Area %
1	15.862	BB	0.2566	1054.52954	63.44704	23.5063
2	18.551	BV	0.3692	3431.63135	142.85867	76.4937
Totals:				4486.16089	206.30572	

(6) *Trans* product from the cyclopropanation catalyzed by mA9A-FeTMPyP2

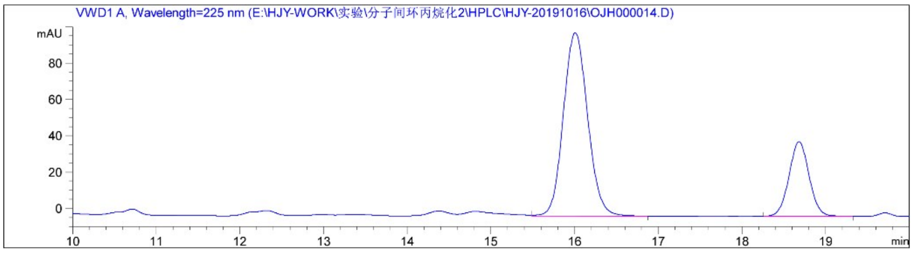

Signal 1: VWD1 A, Wavelength=225 nm

Peak #	RetTime [min]	Type	Width [min]	Area [mAU*s]	Height [mAU]	Area %
1	16.007	VB	0.3207	2046.27869	101.11288	74.8640
2	18.683	BB	0.2575	687.04791	41.13651	25.1360
Totals:				2733.32660	142.24939	

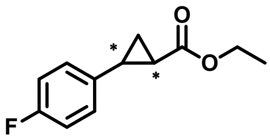

(1) Racemic *trans* product catalyzed by FeTMPyP4

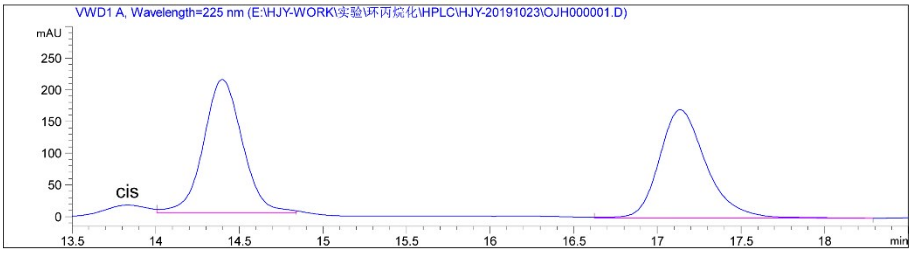

Signal 1: VWD1 A, Wavelength=225 nm

Peak #	RetTime [min]	Type	Width [min]	Area [mAU*s]	Height [mAU]	Area %
1	14.396	MM	0.2711	3429.20825	210.82144	50.6960
2	17.134	VB	0.2963	3335.04468	170.84622	49.3040

Totals: 6764.25293 381.66766

(2) Racemic *trans* product catalyzed by FeTMPyP3

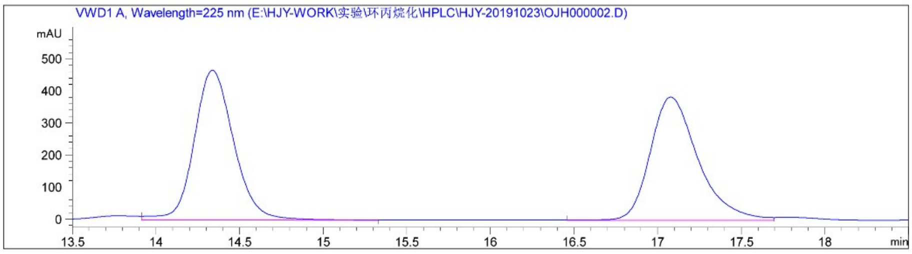

Signal 1: VWD1 A, Wavelength=225 nm

Peak #	RetTime [min]	Type	Width [min]	Area [mAU*s]	Height [mAU]	Area %
1	14.336	VB	0.2539	7726.88379	467.84775	50.2703
2	17.077	FM	0.3314	7643.77783	384.45569	49.7297

Totals: 1.53707e4 852.30344

(3) Racemic *trans* product catalyzed by FeTMPyP2

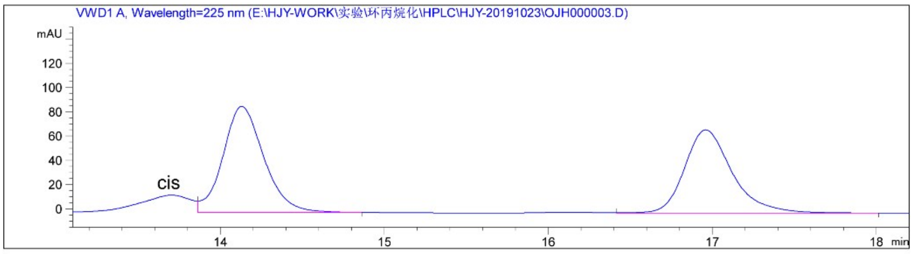

Signal 1: VWD1 A, Wavelength=225 nm

Peak #	RetTime [min]	Type	Width [min]	Area [mAU*s]	Height [mAU]	Area %
1	14.131	VB	0.2636	1512.62354	87.19427	52.1810
2	16.959	VB	0.3074	1386.17859	68.52414	47.8190

Totals: 2898.80212 155.71841

(4) *Trans* product from the cyclopropanation catalyzed by mA9A-FeTMPyP4

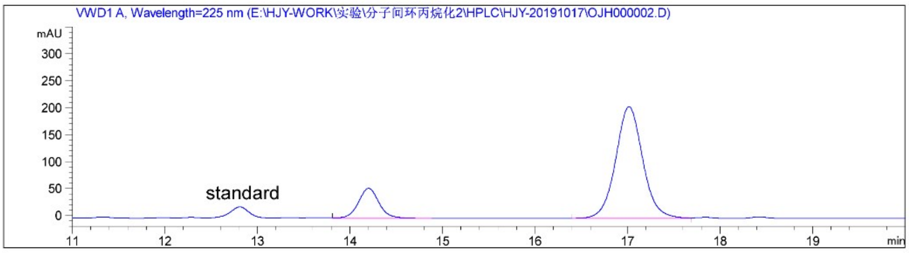

Signal 1: VWD1 A, Wavelength=225 nm

Peak #	RetTime [min]	Type	Width [min]	Area [mAU*s]	Height [mAU]	Area %
1	14.202	VB	0.2396	874.29047	55.82927	17.1389
2	17.016	BV	0.3121	4226.92725	207.41016	82.8611
Totals:				5096.86591	263.24512	

(5) *Trans* product from the cyclopropanation catalyzed by mA9A-FeTMPyP3

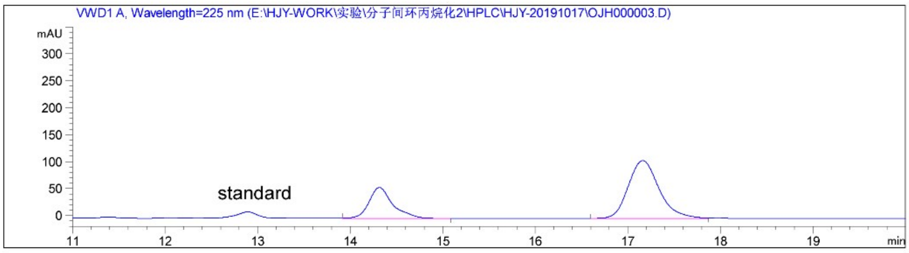

Signal 1: VWD1 A, Wavelength=225 nm

Peak #	RetTime [min]	Type	Width [min]	Area [mAU*s]	Height [mAU]	Area %
1	14.316	VB	0.2650	1031.82813	57.43111	29.3377
2	17.161	BV	0.3605	2485.24097	107.33364	70.6623
Totals:				3517.06909	164.76475	

(6) *Trans* product from the cyclopropanation catalyzed by mA9A-FeTMPyP2

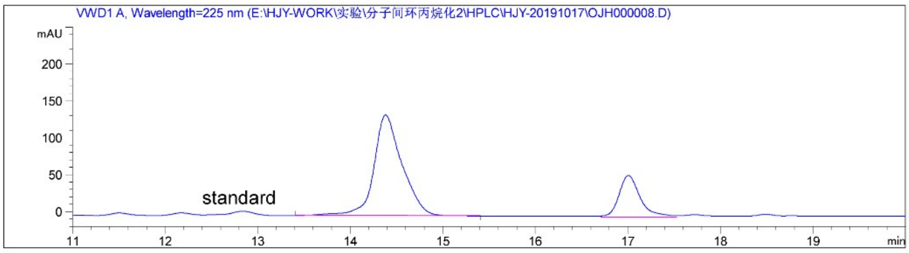

Signal 1: VWD1 A, Wavelength=225 nm

Peak #	RetTime [min]	Type	Width [min]	Area [mAU*s]	Height [mAU]	Area %
1	14.460	VB	0.3152	2623.39624	117.16426	80.2797
2	17.225	BV	0.2244	644.42249	43.71943	19.7203
Totals:				3267.81873	160.88369	

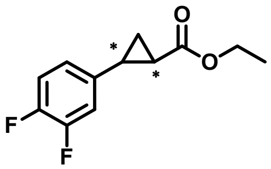

(1) Racemic *trans* product catalyzed by FeTMPyP4

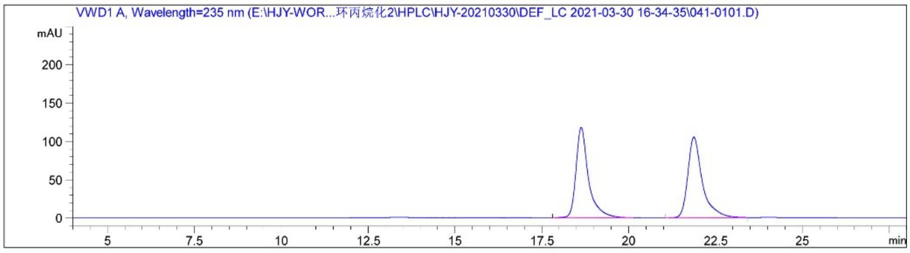

Signal 1: VWD1 A, Wavelength=235 nm

Peak #	RetTime [min]	Type	Width [min]	Area [mAU*s]	Height [mAU]	Area %
1	18.633	BB	0.3744	2981.03467	118.15169	49.2549
2	21.876	BB	0.4324	3071.22632	105.16261	50.7451
Totals:				6052.26099	223.31429	

(2) Racemic *trans* product catalyzed by FeTMPyP3

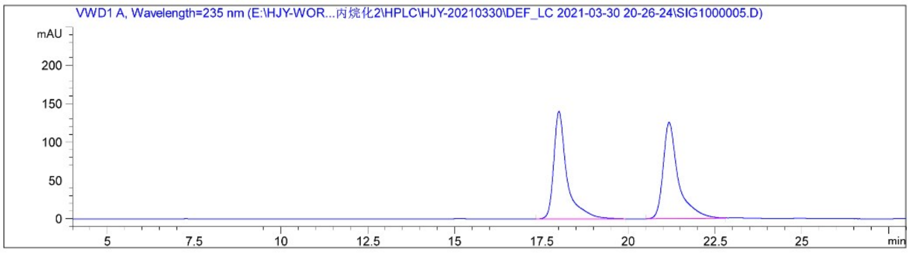

Signal 1: VWD1 A, Wavelength=235 nm

Peak #	RetTime [min]	Type	Width [min]	Area [mAU*s]	Height [mAU]	Area %
1	18.004	BB	0.3844	3712.28394	140.29503	49.3660
2	21.177	BB	0.4430	3807.63623	125.42380	50.6340
Totals:				7519.92017	265.71883	

(3) Racemic *trans* product catalyzed by FeTMPyP2

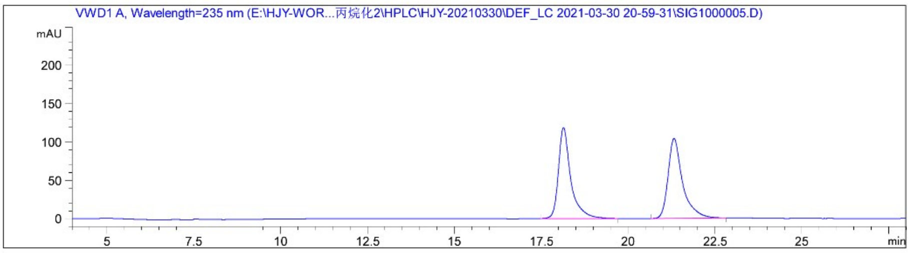

Signal 1: VWD1 A, Wavelength=235 nm

Peak #	RetTime [min]	Type	Width [min]	Area [mAU*s]	Height [mAU]	Area %
1	18.142	BB	0.3658	2957.25635	118.91586	49.2929
2	21.323	BB	0.4309	3042.10400	104.17883	50.7071
Totals:				5999.36035	223.09469	

(4) *Trans* product from the cyclopropanation catalyzed by mA9A-FeTMPyP4

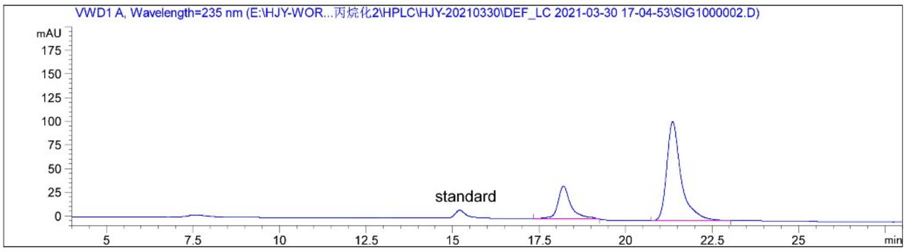

Signal 1: VWD1 A, Wavelength=235 nm

Peak #	RetTime [min]	Type	Width [min]	Area [mAU*s]	Height [mAU]	Area %
1	18.203	MM	0.4314	896.16675	34.62307	22.7724
2	21.358	BB	0.4290	3039.14722	104.67245	77.2276
Totals:				3935.31396	139.29552	

(5) *Trans* product from the cyclopropanation catalyzed by mA9A-FeTMPyP3

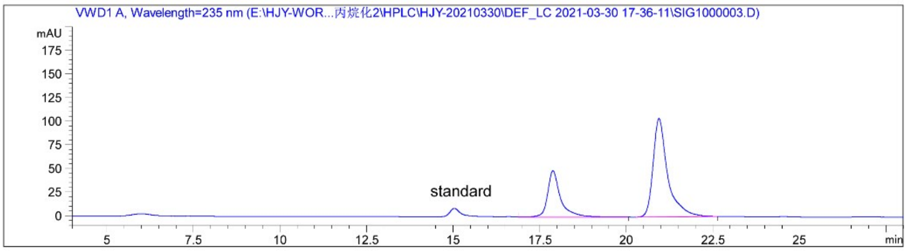

Signal 1: VWD1 A, Wavelength=235 nm

Peak #	RetTime [min]	Type	Width [min]	Area [mAU*s]	Height [mAU]	Area %
1	17.883	MM	0.4409	1297.94507	49.06865	29.8083
2	20.946	BB	0.4341	3056.36743	104.14095	70.1917
Totals:				4354.31250	153.20960	

(6) *Trans* product from the cyclopropanation catalyzed by mA9A-FeTMPyP2

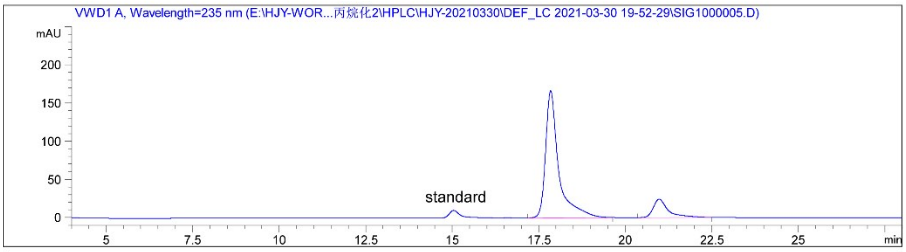

Signal 1: VWD1 A, Wavelength=235 nm

Peak #	RetTime [min]	Type	Width [min]	Area [mAU*s]	Height [mAU]	Area %
1	17.843	BB	0.3843	4409.87988	166.69745	85.6408
2	20.980	BB	0.4468	739.39587	24.19450	14.3592
Totals:				5149.27576	190.89195	

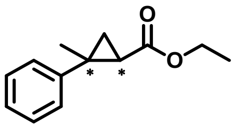

(1) Racemic *trans* product catalyzed by FeTMPyP4

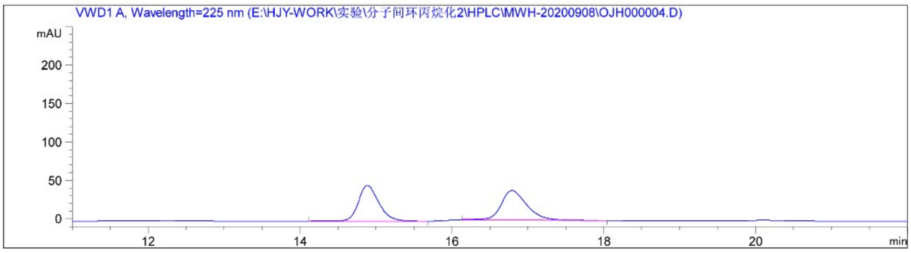

Signal 1: VWD1 A, Wavelength=225 nm

Peak #	RetTime [min]	Type	Width [min]	Area [mAU*s]	Height [mAU]	Area %
1	14.888	BB	0.2871	876.25323	46.46601	49.3497
2	16.793	MM	0.3916	899.34650	38.27814	50.6503
Totals:				1775.59973	84.74415	

(2) Racemic *trans* product catalyzed by FeTMPyP3

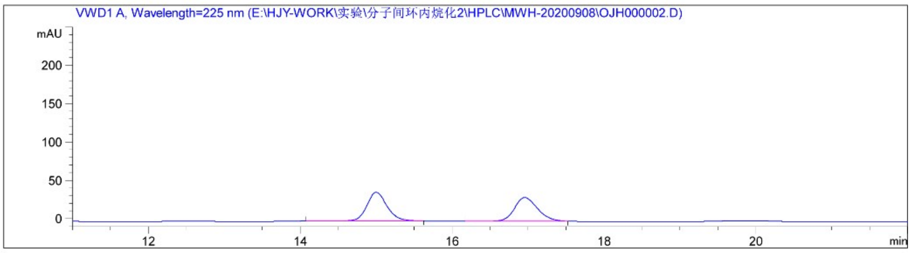

Signal 1: VWD1 A, Wavelength=225 nm

Peak #	RetTime [min]	Type	Width [min]	Area [mAU*s]	Height [mAU]	Area %
1	15.001	MM	0.3079	691.40973	37.42679	51.5734
2	16.959	MM	0.3505	649.22241	30.86921	48.4266
Totals:				1340.63214	68.29600	

(3) Racemic *trans* product catalyzed by FeTMPyP2

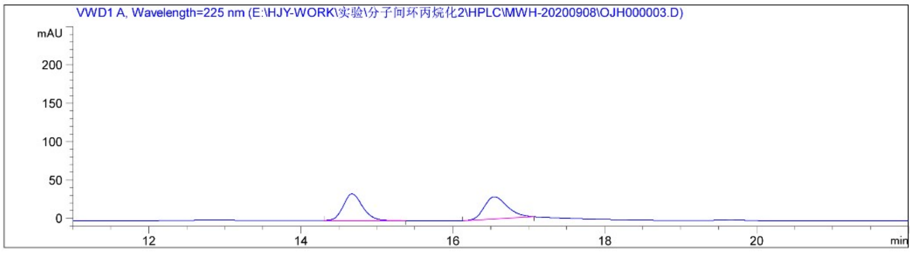

Signal 1: VWD1 A, Wavelength=225 nm

Peak #	RetTime [min]	Type	Width [min]	Area [mAU*s]	Height [mAU]	Area %
1	14.676	BB	0.2744	630.75702	34.99970	50.4532
2	16.550	MM	0.3541	619.42499	29.15617	49.5468
Totals:				1250.18201	64.15587	

(4) *Trans* product from the cyclopropanation catalyzed by mA9A-FeTMPyP4

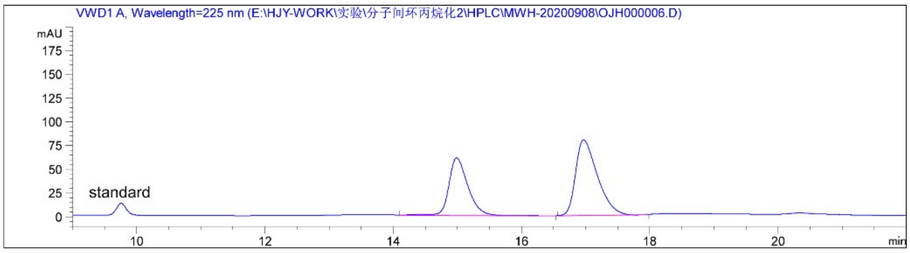

Signal 1: VWD1 A, Wavelength=225 nm

Peak #	RetTime [min]	Type	Width [min]	Area [mAU*s]	Height [mAU]	Area %
1	15.382	FM	0.3560	1303.87903	61.04063	40.9001
2	17.361	MM	0.3939	1884.08057	79.70947	59.0999
Totals:				3187.95959	140.75010	

(5) *Trans* product from the cyclopropanation catalyzed by mA9A-FeTMPyP3

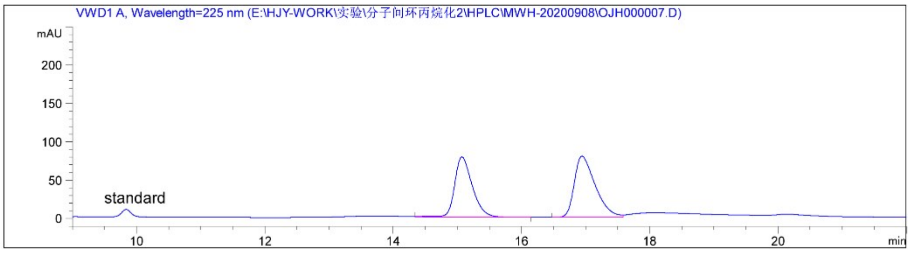

Signal 1: VWD1 A, Wavelength=225 nm

Peak #	RetTime [min]	Type	Width [min]	Area [mAU*s]	Height [mAU]	Area %
1	15.348	MM	0.3336	1569.93311	78.42530	45.1246
2	17.314	BV	0.3620	1909.17249	79.87239	54.8754
Totals:				3479.10559	158.29769	

(6) *Trans* product from the cyclopropanation catalyzed by mA9A-FeTMPyP2

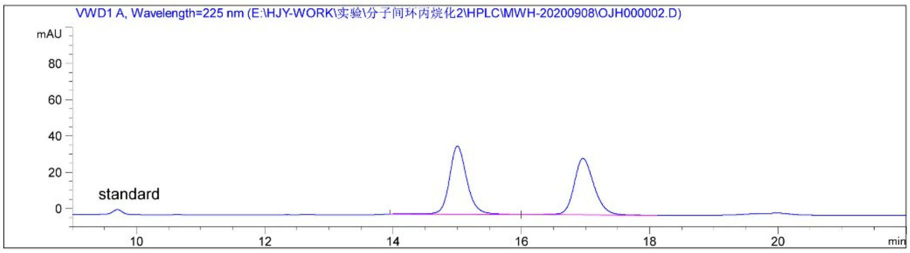

Signal 1: VWD1 A, Wavelength=225 nm

Peak #	RetTime [min]	Type	Width [min]	Area [mAU*s]	Height [mAU]	Area %
1	15.001	MF	0.3198	724.20697	37.74258	51.6815
2	16.959	FM	0.3618	677.08167	31.18903	48.3185
Totals:				1401.28864	68.93161	

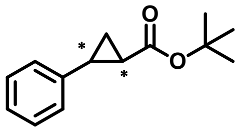

(1) Racemic *trans* product catalyzed by FeTMPyP4

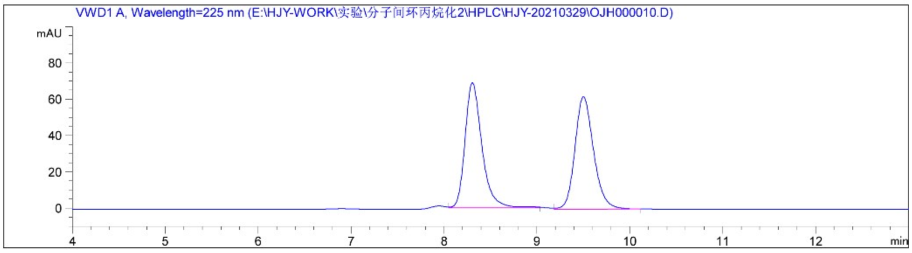

Signal 1: VWD1 A, Wavelength=225 nm

Peak #	RetTime [min]	Type	Width [min]	Area [mAU*s]	Height [mAU]	Area %
1	8.305	FM	0.2117	876.82294	69.01480	50.3834
2	9.500	VB	0.2137	863.47778	61.92261	49.6166

Totals: 1740.30072 130.93741

(2) Racemic *trans* product catalyzed by FeTMPyP3

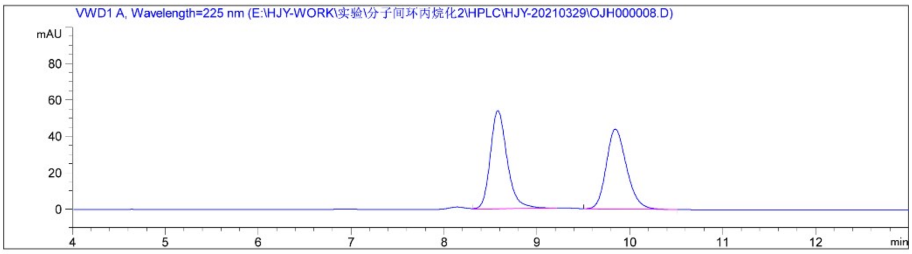

Signal 1: VWD1 A, Wavelength=225 nm

Peak #	RetTime [min]	Type	Width [min]	Area [mAU*s]	Height [mAU]	Area %
1	8.874	VB	0.2045	722.56366	53.89443	50.3919
2	10.223	BB	0.2487	711.32343	43.93259	49.6081

Totals: 1433.88708 97.82702

(3) Racemic *trans* product catalyzed by FeTMPyP2

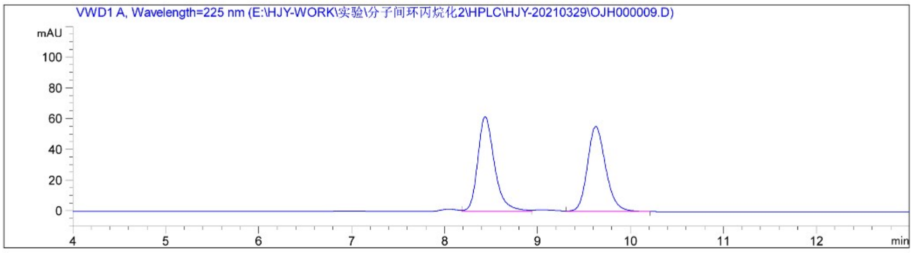

Signal 1: VWD1 A, Wavelength=225 nm

Peak #	RetTime [min]	Type	Width [min]	Area [mAU*s]	Height [mAU]	Area %
1	8.436	VB	0.1908	777.01953	61.62082	50.6113
2	9.626	VB	0.2093	758.24988	55.37621	49.3887

Totals: 1535.26941 116.99703

(4) *Trans* product from the cyclopropanation catalyzed by mA9A-FeTMPyP4

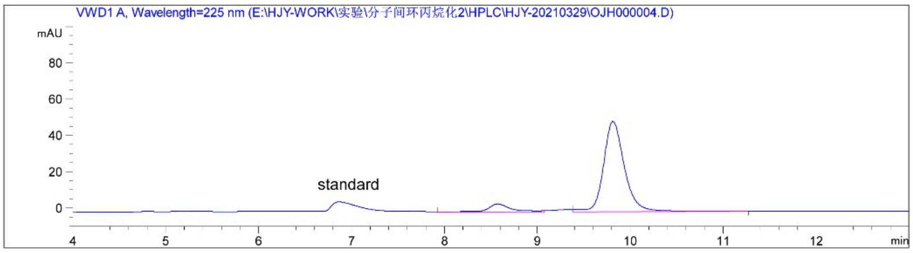

Signal 1: VWD1 A, Wavelength=225 nm

Peak #	RetTime [min]	Type	Width [min]	Area [mAU*s]	Height [mAU]	Area %
1	8.573	MF	0.2953	77.96986	4.40032	9.0409
2	9.813	FM	0.2628	784.43921	49.74258	90.9591
Totals:				862.40907	54.14290	

(5) *Trans* product from the cyclopropanation catalyzed by mA9A-FeTMPyP3

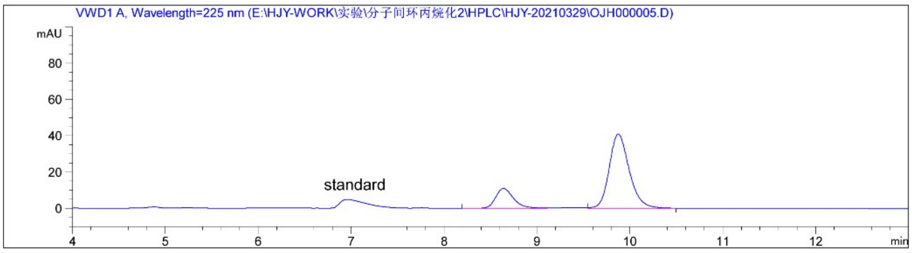

Signal 1: VWD1 A, Wavelength=225 nm

Peak #	RetTime [min]	Type	Width [min]	Area [mAU*s]	Height [mAU]	Area %
1	8.638	BB	0.2073	151.08315	11.07085	19.7433
2	9.874	VB	0.2288	614.15540	40.98235	80.2567
Totals:				765.23854	52.05319	

(6) *Trans* product from the cyclopropanation catalyzed by mA9A-FeTMPyP2

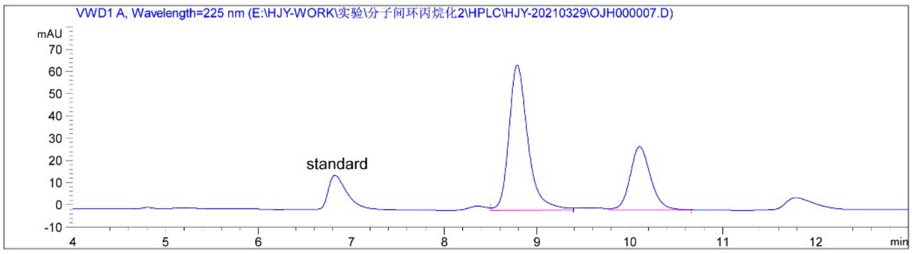

Signal 1: VWD1 A, Wavelength=225 nm

Peak #	RetTime [min]	Type	Width [min]	Area [mAU*s]	Height [mAU]	Area %
1	8.785	VB	0.2137	929.03815	65.43421	67.8835
2	10.103	VB	0.2351	439.53946	28.53935	32.1165
Totals:				1368.57761	93.97356	

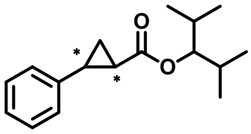

(1) Racemic *trans* product catalyzed by FeTMPyP4

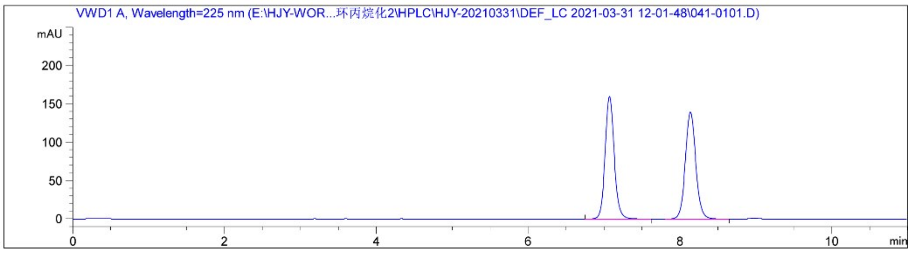

Signal 1: VWD1 A, Wavelength=225 nm

Peak #	RetTime [min]	Type	Width [min]	Area [mAU*s]	Height [mAU]	Area %
1	7.072	BB	0.1286	1343.86194	159.94370	49.9669
2	8.139	BB	0.1473	1345.64233	139.78761	50.0331
Totals:				2689.50427	299.73131	

(2) Racemic *trans* product catalyzed by FeTMPyP3

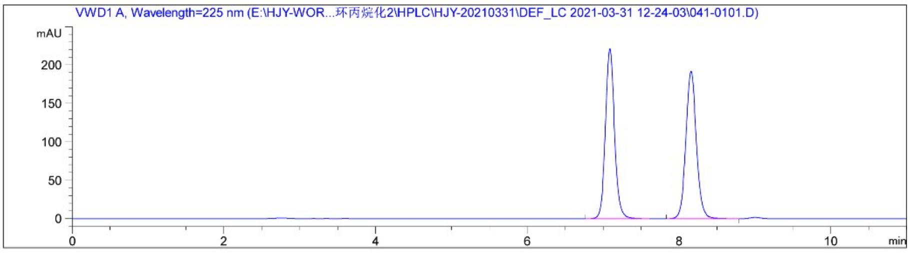

Signal 1: VWD1 A, Wavelength=225 nm

Peak #	RetTime [min]	Type	Width [min]	Area [mAU*s]	Height [mAU]	Area %
1	7.088	BB	0.1284	1856.69983	221.48999	49.9503
2	8.158	BB	0.1494	1860.39575	192.16177	50.0497
Totals:				3717.09558	413.65176	

(3) Racemic *trans* product catalyzed by FeTMPyP2

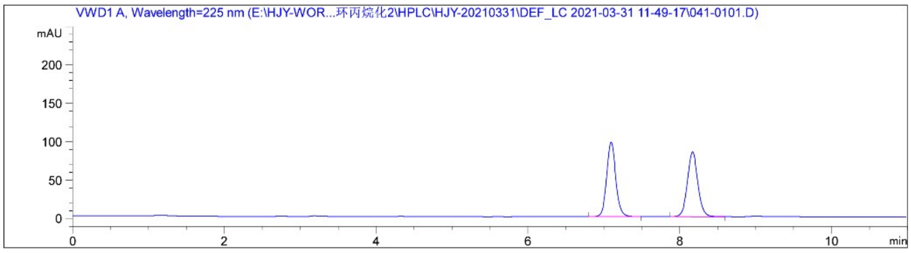

Signal 1: VWD1 A, Wavelength=225 nm

Peak #	RetTime [min]	Type	Width [min]	Area [mAU*s]	Height [mAU]	Area %
1	7.096	BB	0.1282	812.85834	97.11717	50.0823
2	8.169	VB	0.1485	810.18530	84.33962	49.9177
Totals:				1623.04364	181.45679	

(4) *Trans* product from the cyclopropanation catalyzed by mA9A-FeTMPyP4

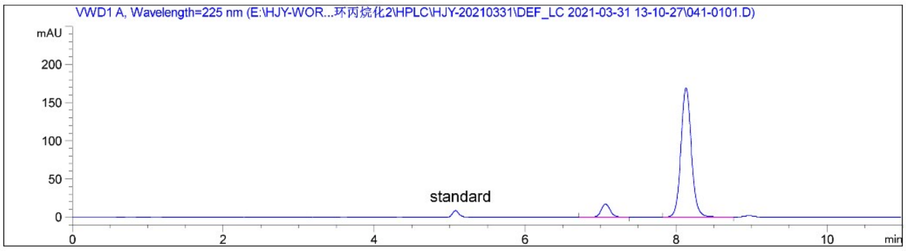

Signal 1: VWD1 A, Wavelength=225 nm

Peak #	RetTime [min]	Type	Width [min]	Area [mAU*s]	Height [mAU]	Area %
1	7.066	BB	0.1289	145.37338	17.25607	8.1401
2	8.131	BB	0.1478	1640.51172	169.65576	91.8599
Totals:				1785.88510	186.91184	

(5) *Trans* product from the cyclopropanation catalyzed by mA9A-FeTMPyP3

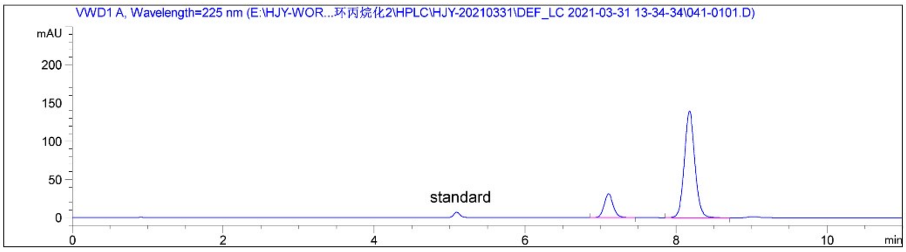

Signal 1: VWD1 A, Wavelength=225 nm

Peak #	RetTime [min]	Type	Width [min]	Area [mAU*s]	Height [mAU]	Area %
1	7.105	BB	0.1284	262.00031	31.24370	16.1732
2	8.177	BB	0.1497	1357.96790	139.81061	83.8268
Totals:				1619.96820	171.05431	

(6) *Trans* product from the cyclopropanation catalyzed by mA9A-FeTMPyP2

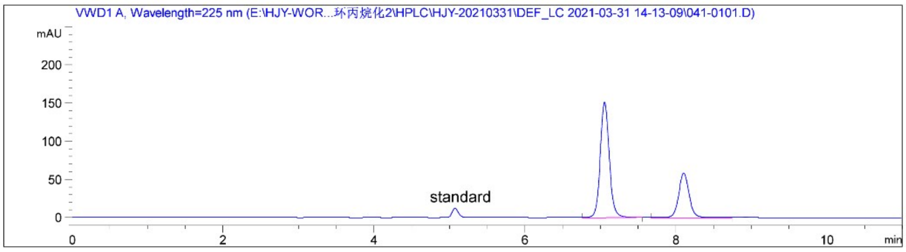

Signal 1: VWD1 A, Wavelength=225 nm

Peak #	RetTime [min]	Type	Width [min]	Area [mAU*s]	Height [mAU]	Area %
1	7.056	BB	0.1278	1260.31360	151.22862	69.0247
2	8.104	MM	0.1616	565.57422	58.33767	30.9753
Totals:				1825.88782	209.56629	

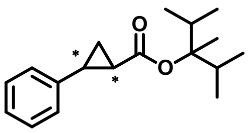

(1) Racemic *trans* product catalyzed by FeTMPyP4

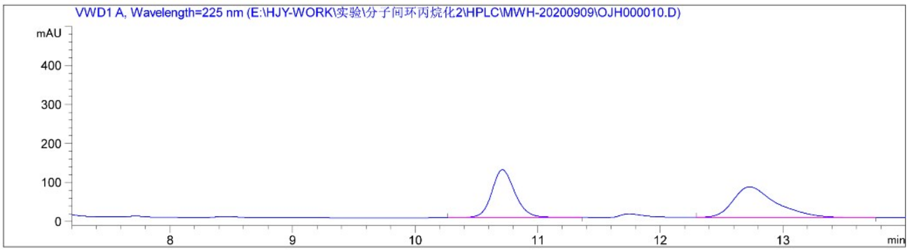

Signal 1: VWD1 A, Wavelength=225 nm

Peak #	RetTime [min]	Type	Width [min]	Area [mAU*s]	Height [mAU]	Area %
1	10.713	MM	0.2456	1845.59424	125.22459	48.8721
2	12.730	MM	0.4078	1930.78052	78.90295	51.1279
Totals:				3776.37476	204.12755	

(2) Racemic *trans* product catalyzed by FeTMPyP3

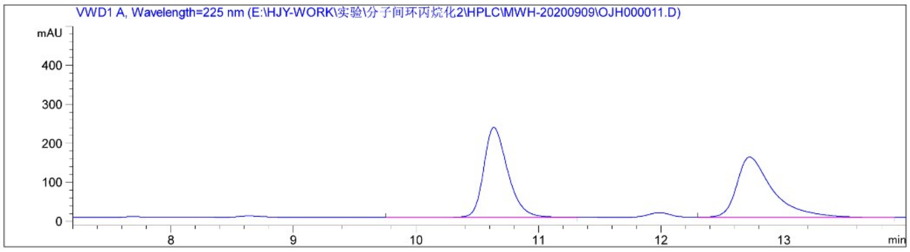

Signal 1: VWD1 A, Wavelength=225 nm

Peak #	RetTime [min]	Type	Width [min]	Area [mAU*s]	Height [mAU]	Area %
1	10.785	BB	0.2124	2421.90723	172.01741	47.5126
2	12.908	VB	0.3132	2675.48975	126.80769	52.4874
Totals:				5097.39697	298.82510	

(3) Racemic *trans* product catalyzed by FeTMPyP2

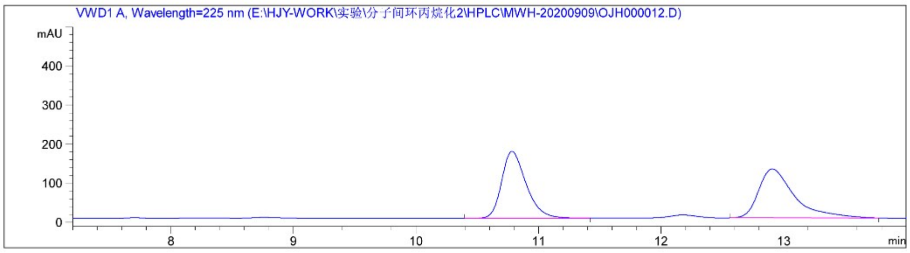

Signal 1: VWD1 A, Wavelength=225 nm

Peak #	RetTime [min]	Type	Width [min]	Area [mAU*s]	Height [mAU]	Area %
1	10.713	MM	0.2330	1747.22778	124.99615	48.2356
2	12.730	MM	0.3989	1875.04919	78.34483	51.7644
Totals:				3622.27698	203.34097	

(4) *Trans* product from the cyclopropanation catalyzed by mA9A-FeTMPyP4

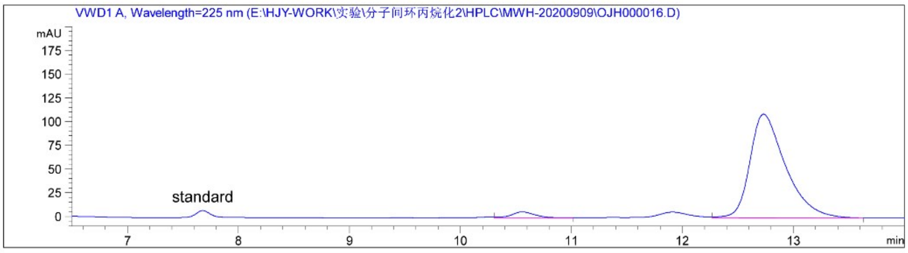

Signal 1: VWD1 A, Wavelength=225 nm

Peak #	RetTime [min]	Type	Width [min]	Area [mAU*s]	Height [mAU]	Area %
1	10.556	MM	0.2902	113.22777	6.50344	4.4336
2	12.732	VB	0.3317	2440.62915	109.42220	95.5664
Totals:				2553.85692	115.92564	

(5) *Trans* product from the cyclopropanation catalyzed by mA9A-FeTMPyP3

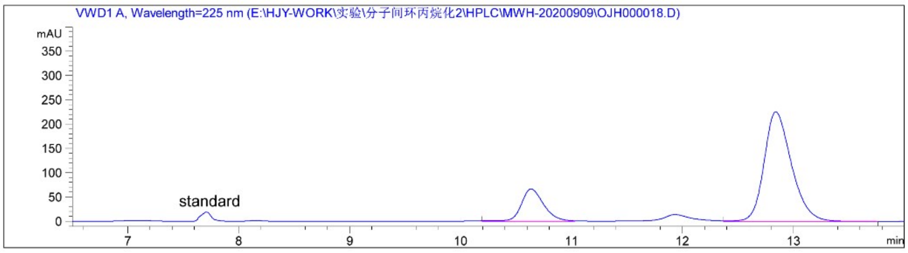

Signal 1: VWD1 A, Wavelength=225 nm

Peak #	RetTime [min]	Type	Width [min]	Area [mAU*s]	Height [mAU]	Area %
1	10.448	MM	0.2120	826.29364	64.94648	18.1963
2	12.554	VV	0.2501	3714.70581	225.94635	81.8037
Totals:				4540.99945	290.89283	

(6) *Trans* product from the cyclopropanation catalyzed by mA9A-FeTMPyP2

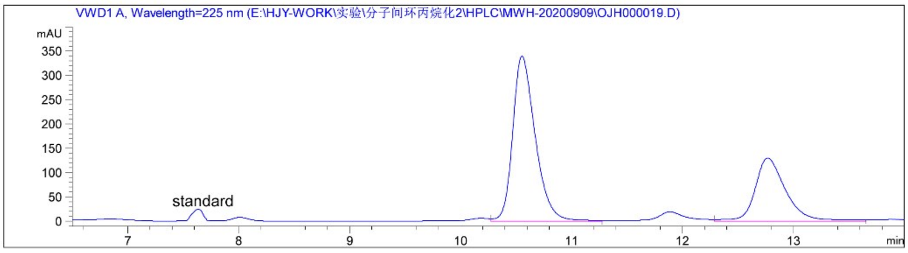

Signal 1: VWD1 A, Wavelength=225 nm

Peak #	RetTime [min]	Type	Width [min]	Area [mAU*s]	Height [mAU]	Area %
1	10.403	VB	0.2001	4474.54492	340.25177	65.5142
2	12.460	VV	0.2695	2355.33862	130.09409	34.4858
Totals:				6829.88354	470.34586	

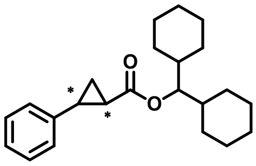

(1) Racemic *trans* product catalyzed by FeTMPyP4

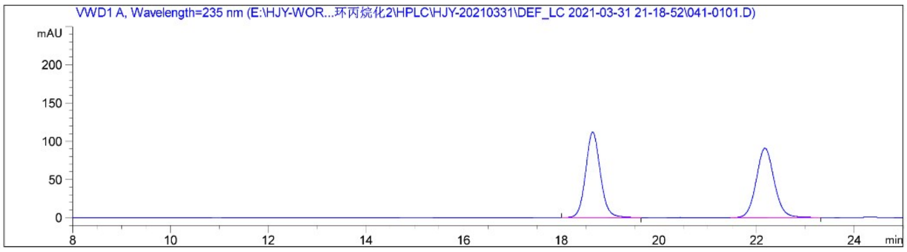

Signal 1: VWD1 A, Wavelength=235 nm

Peak #	RetTime [min]	Type	Width [min]	Area [mAU*s]	Height [mAU]	Area %
1	18.644	BB	0.3210	2358.11255	112.14309	50.0232
2	22.168	BB	0.3979	2355.92896	91.08597	49.9768
Totals:				4714.04150	203.22906	

(2) Racemic *trans* product catalyzed by FeTMPyP3

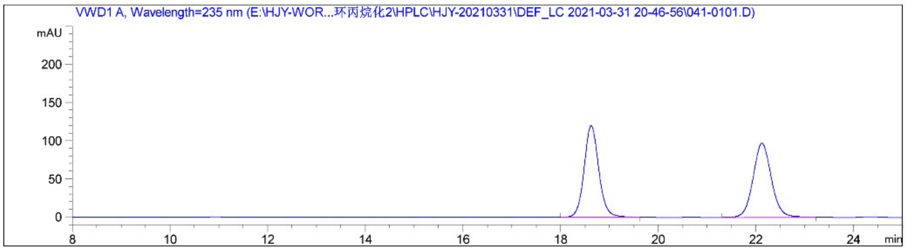

Signal 1: VWD1 A, Wavelength=235 nm

Peak #	RetTime [min]	Type	Width [min]	Area [mAU*s]	Height [mAU]	Area %
1	18.623	BB	0.3198	2511.68237	120.07256	49.8477
2	22.119	BB	0.3981	2527.03516	97.14995	50.1523
Totals:				5038.71753	217.22250	

(3) Racemic *trans* product catalyzed by FeTMPyP2

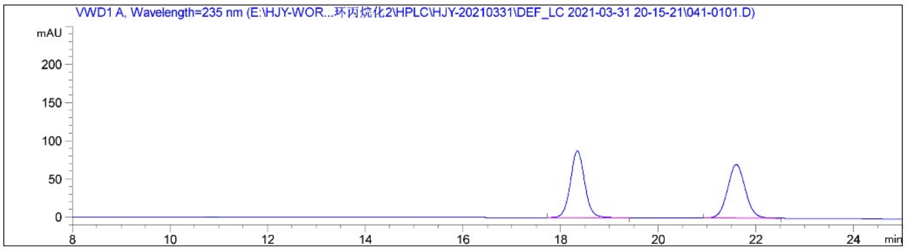

Signal 1: VWD1 A, Wavelength=235 nm

Peak #	RetTime [min]	Type	Width [min]	Area [mAU*s]	Height [mAU]	Area %
1	18.341	BB	0.3143	1803.07214	87.62695	50.3330
2	21.594	BB	0.3893	1779.21704	70.45657	49.6670
Totals:				3582.28918	158.08351	

(4) *Trans* product from the cyclopropanation catalyzed by mA9A-FeTMPyP4

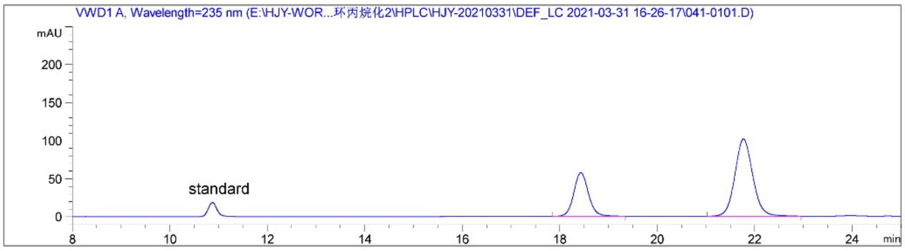

Signal 1: VWD1 A, Wavelength=235 nm

Peak #	RetTime [min]	Type	Width [min]	Area [mAU*s]	Height [mAU]	Area %
1	18.431	BB	0.3212	1203.09607	57.50661	31.7402
2	21.767	BB	0.3899	2587.35376	101.72431	68.2598

Totals: 3790.44983 159.23092

(5) *Trans* product from the cyclopropanation catalyzed by mA9A-FeTMPyP3

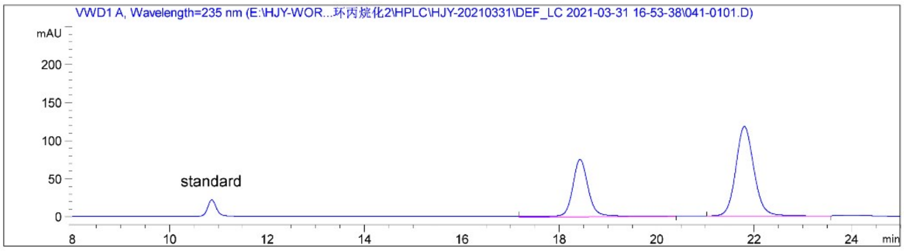

Signal 1: VWD1 A, Wavelength=235 nm

Peak #	RetTime [min]	Type	Width [min]	Area [mAU*s]	Height [mAU]	Area %
1	18.426	MM	0.3949	1795.60107	75.77421	37.2007
2	21.798	MM	0.4298	3031.19507	117.54772	62.7993

Totals: 4826.79614 193.32193

(6) *Trans* product from the cyclopropanation catalyzed by mA9A-FeTMPyP2

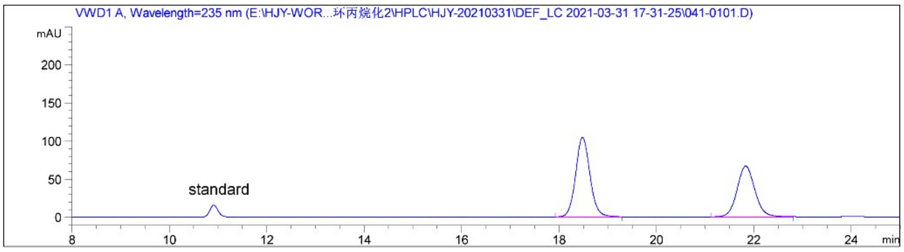

Signal 1: VWD1 A, Wavelength=235 nm

Peak #	RetTime [min]	Type	Width [min]	Area [mAU*s]	Height [mAU]	Area %
1	18.481	BB	0.3144	2135.03052	104.36347	55.7954
2	21.830	BB	0.3903	1691.50549	66.73677	44.2046

Totals: 3826.53601 171.10024

References

1. Y. Li, M. Cheng, J. Hao, C. Wang, G. Jia and C. Li, *Chemical Science*, 2015, **6**, 5578-5585.
2. M. P. Cheng, J. Y. Hao, Y. H. Li, Y. Cheng, G. Q. Jia, J. Zhou and C. Li, *Biochimie*, 2018, **146**, 20-27.
3. T. D. J. Stumpf, M. Steinbach, M. Holtke, G. Heuger, F. Grasemann, R. Frohlich, S. Schindler and R. Gottlich, *Eur J Org Chem*, 2018, **2018**, 5538-5547.
4. A. Rioz-Martinez, J. Oelerich, N. Segaud and G. Roelfes, *Angewandte Chemie-International Edition*, 2016, **55**, 14136-14140.
5. H. B. Mao, A. J. Lin, Y. Shi, Z. J. Mao, X. B. Zhu, W. P. Li, H. W. Hu, Y. X. Cheng and C. J. Zhu, *Angewandte Chemie-International Edition*, 2013, **52**, 6288-6292.
6. P. S. Coelho, E. M. Brustad, A. Kannan and F. H. Arnold, *Science*, 2013, **339**, 307-310.
7. A. Sarkar, D. Formenti, F. Ferretti, C. Kreyenschulte, S. Bartling, K. Junge, M. Beller and F. Ragaini, *Chemical Science*, 2020, **11**, 6217-6221.
8. K. E. Hernandez, H. Renata, R. D. Lewis, S. B. J. Kan, C. Zhang, J. Forte, D. Rozzell, J. A. McIntosh and F. H. Arnold, *Acs Catalysis*, 2016, **6**, 7810-7813.
9. N. Watanabe, H. Matsuda, H. Kuribayashi and S. Hashimoto, *Heterocycles*, 1996, **42**, 537-542.
10. M. P. Doyle, B. D. Brandes, A. P. Kazala, R. J. Pieters, M. B. Jarstfer, L. M. Watkins and C. T. Eagle, *Tetrahedron Lett*, 1990, **31**, 6613-6616.
11. J. A. Ma, L. X. Wang, W. Zhang and Q. L. Zhou, *Tetrahedron-Asymmetr*, 2001, **12**, 2801-2804.
